# Supplementary material for: Typhoid toxin of Salmonella Typhi elicits host antimicrobial response during acute typhoid fever
Source: EMBO Mol Med. 2025 Dec 1;18(1):187–216. doi: 10.1038/s44321-025-00347-8 (PMC12808722; doi:10.1038/s44321-025-00347-8)
Supplement: Supplementary file 1 — Appendix [file 44321_2025_347_MOESM1_ESM.pdf]

Appendix

Appendix Table S1..1-2

Appendix Table S1 – *P* values

Figure	P-value
	**Figure 2**
2B	Unt vs WT *p*=0.0002, unt vs HQ *p*=0.9703, WT vs HQ *p*=0.0003,
2C	Unt vs WT *p*<0.0001, unt vs HQ *p*=0.0041, WT vs HQ *p*<0.0001
2D	Unt vs WT *p*=0.0274, unt vs HQ *p*=0.9831, WT vs HQ *p*=0.0337
2E	Unt vs WT *p*<0.0001, unt vs HQ *p*=0.0001, WT vs HQ *p*<0.0001
2G	WT vs *ΔcdtB p*=0.0121, WT vs *ΔcdtB* pCdtB *p*=0.1152, *ΔcdtB* vs *ΔcdtB* pCdtB *p*=0.0013
2H	WT vs *ΔcdtB p*=0.0364, WT vs *ΔcdtB* pCdtB *p*=0.9982, *ΔcdtB* vs *ΔcdtB* pCdtB *p*=0.039
	**Figure 3**
3B	*p*=0.0132
3C	*p*=0.0013
3E	unt vs LYZ *p*=0.0011, unt vs LFN *p*=0.0689, unt vs LYZ/LFN *p*<0.0001, unt vs LYZ/EDTA *p*<0.0001
3H	LYZ *p*<0.0001, LFN *p*=0.0895, LYZ/LFN *p*<0.0001, LYZ/EDTA *p*<0.0001, rec LYZ *p*<0.0001
	**Figure 4**
4B	LYZ *p*<0.0001, LFN *p*=0.0268, LYZLFN *p*<0.0001, rLYZ *p*<0.0001
4C	LYZ *p*=0.0046, LFN *p*=0.4577, LYZLFN *p*=0.9249, rLYZ *p*=0.0067
4F	LYZ 1000µg *p*<0.0001, LYZ 100µg *p*<0.0001, LYZ 10µg *p*=0.0016, LYZ 1µg *p*=0.0954, LYZ 0.1µg *p*=0.0813, denat. LYZ *p*>0.9999
4G	*p*=0.0257
4H	2h *p*=0.9623, 24h *p*=0.0479
	**Figure 5**
5C	*p*<0.0001
5D	*p*=0.0374
	**Figure 6**
6B	*p*=0.0143
6C	*p*=0.0001
6F	CCCP *p*<0.0001, MitoQ *p*<0.0001, NADC *p*=0.256, DPI *p*=0.0005, exog LYZ *p*<0.0001

6G	CCCP *p*=0.0076, MitoQ *p*=0.002, NADC *p*=0.0274, DPI *p*=0.1313, exog LYZ *p*=0.4529
6I	unt *p*=0.9908, TxWT *p*=0.0335
**Figure EV1**	
EV1B	*p*=0.1551
EV1C	*p*=0.0016
EV1D	1h (*p*=0.9627), 2h (*p*=0.8889) and 4h (*p*=7768) (n=3)
**Figure EV2**	
EV2A	unt vs ETP *p*=0.4297, unt vs FBS *p*=0.9285, ETP vs FBS *p*=0.3356
EV2B	*p*=0.0233
EV2D	LYZ p<0.0001, LFN p=0.373, LYZ+LFN p<0.0001, LYZ+EDTA p<0.0001
EV2E	LYZ p=0.0009, LFN p>0.9999, LYZ+LFN p=0.0002, LYZ+EDTA p<0.0001
**Figure EV3**	
EV3E	*p*=0.1977
