# Supplementary figures and images for: Typhoid toxin of Salmonella Typhi elicits host antimicrobial response during acute typhoid fever
Source: EMBO Mol Med. 2025 Dec 1;18(1):187–216. doi: 10.1038/s44321-025-00347-8 (PMC12808722; doi:10.1038/s44321-025-00347-8)

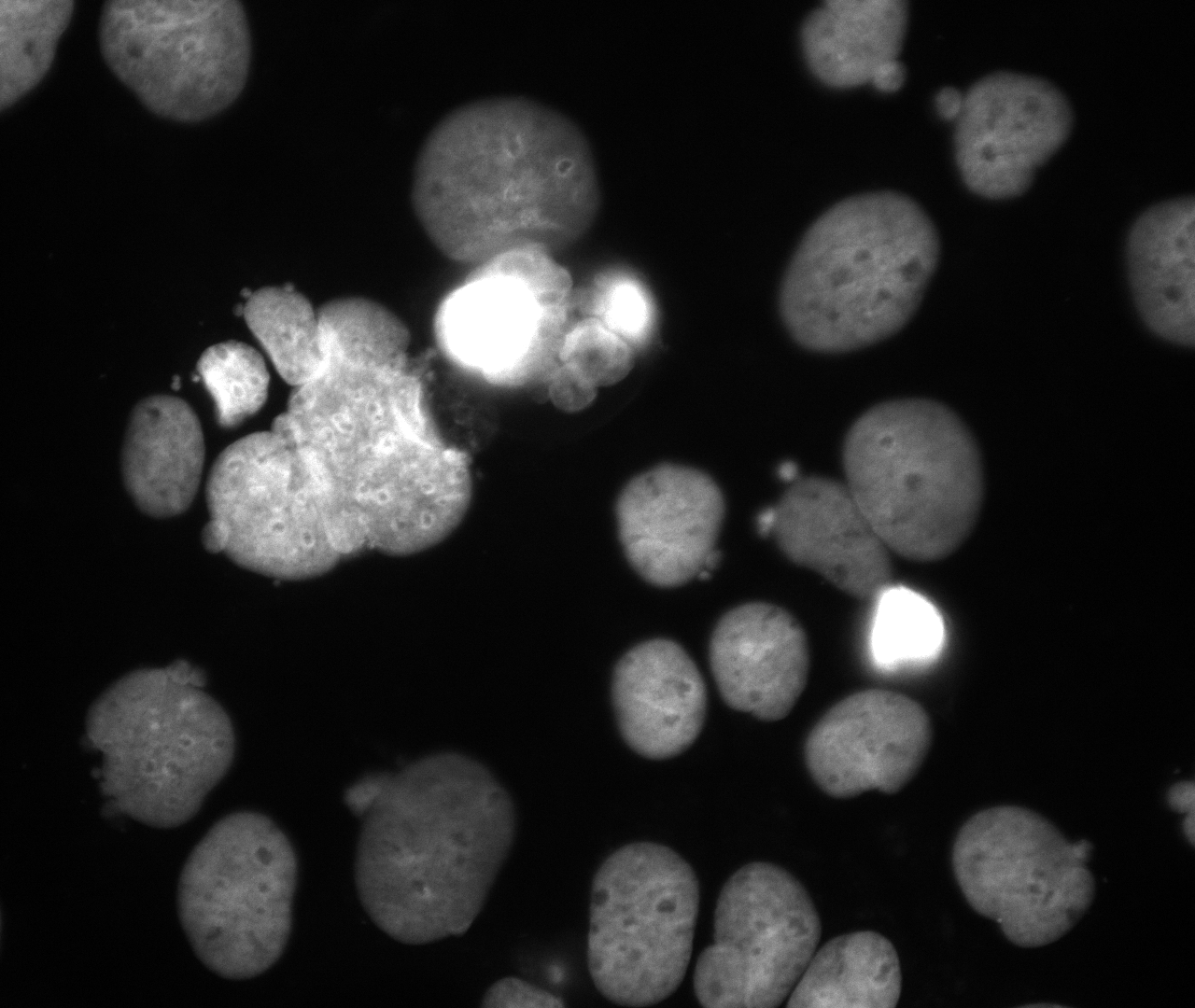

Supplement: Supplementary file 5 — Source data Fig. 2 [file 44321_2025_347_MOESM5_ESM.zip › SD for Fig 2/2F/2F (top panel).tif]

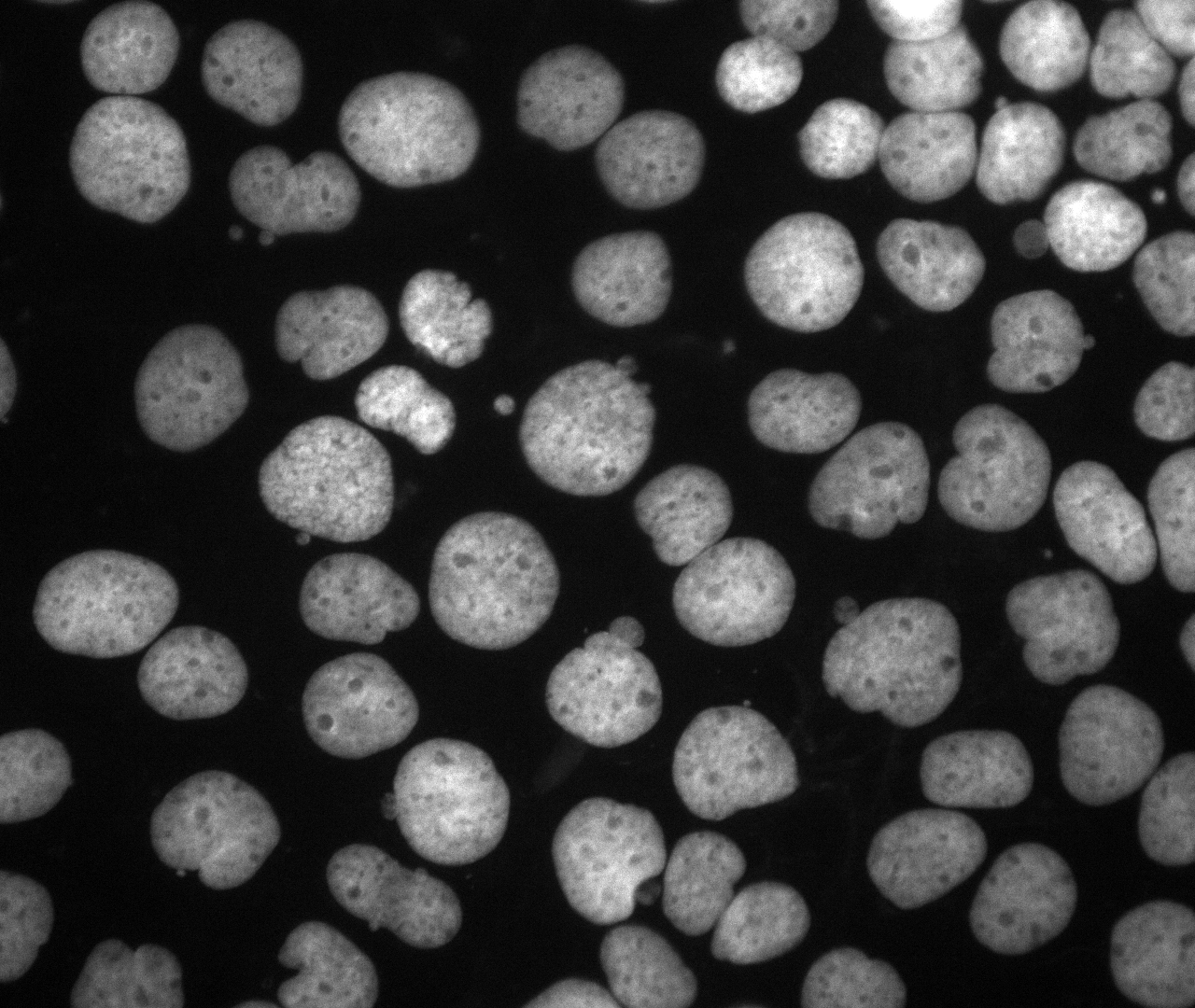

Supplement: Supplementary file 5 — Source data Fig. 2 [file 44321_2025_347_MOESM5_ESM.zip › SD for Fig 2/2F/2F (bottom panel).tif]

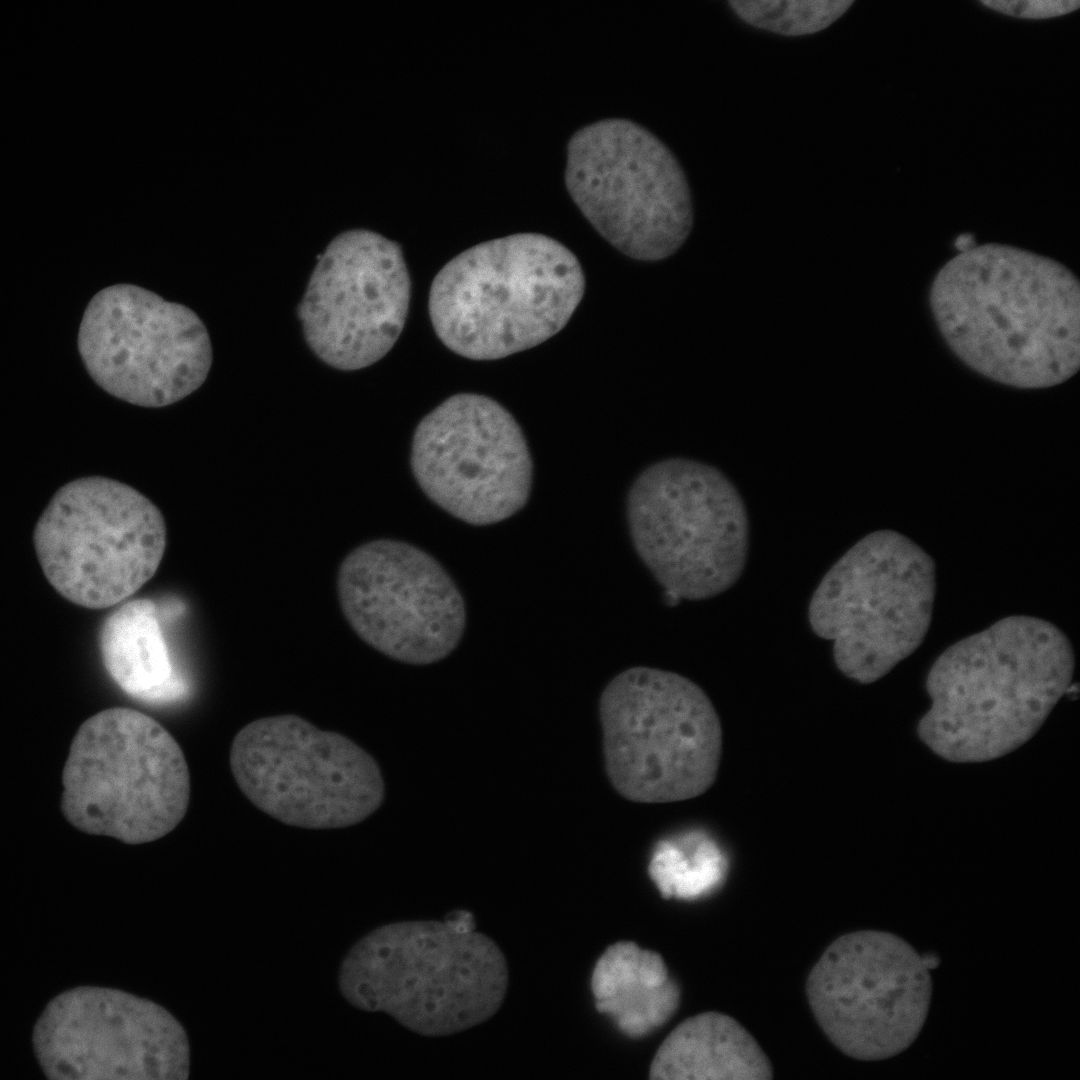

Supplement: Supplementary file 5 — Source data Fig. 2 [file 44321_2025_347_MOESM5_ESM.zip › SD for Fig 2/2A/2A (right panel) /2A (HQ).tif]

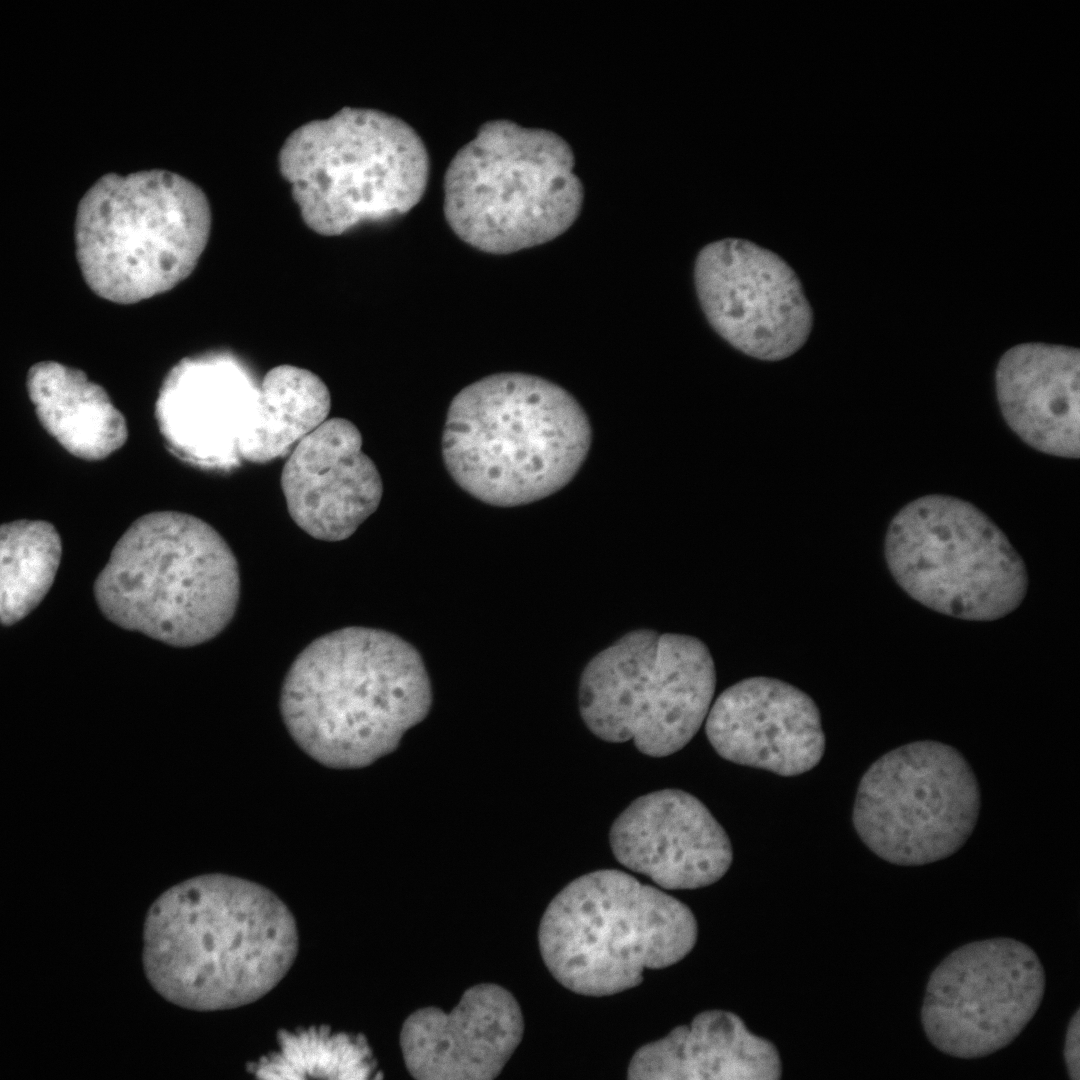

Supplement: Supplementary file 5 — Source data Fig. 2 [file 44321_2025_347_MOESM5_ESM.zip › SD for Fig 2/2A/2A (left panel)/2A (unt).tif]

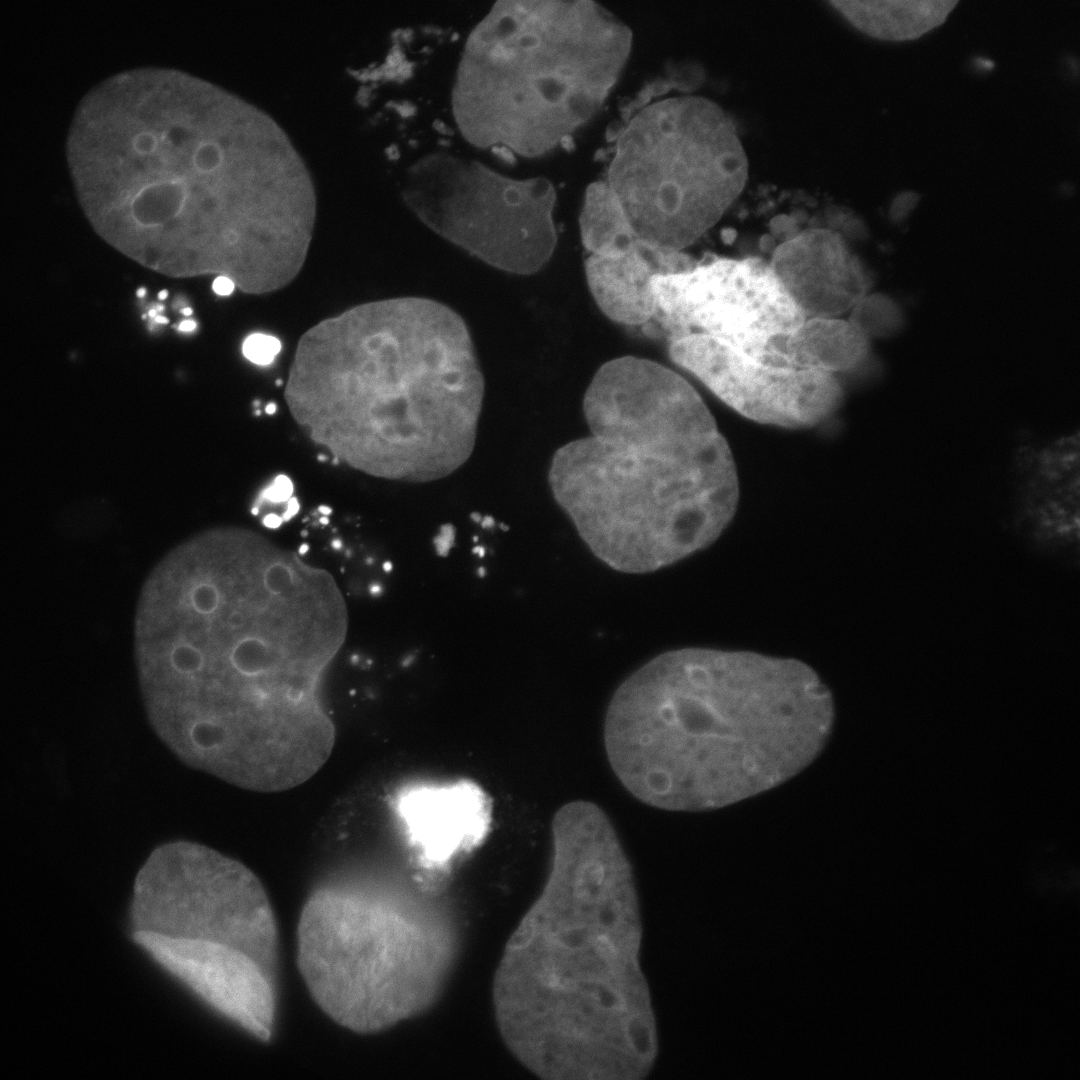

Supplement: Supplementary file 5 — Source data Fig. 2 [file 44321_2025_347_MOESM5_ESM.zip › SD for Fig 2/2A/2A (middle panel)/2A (TT).tif]

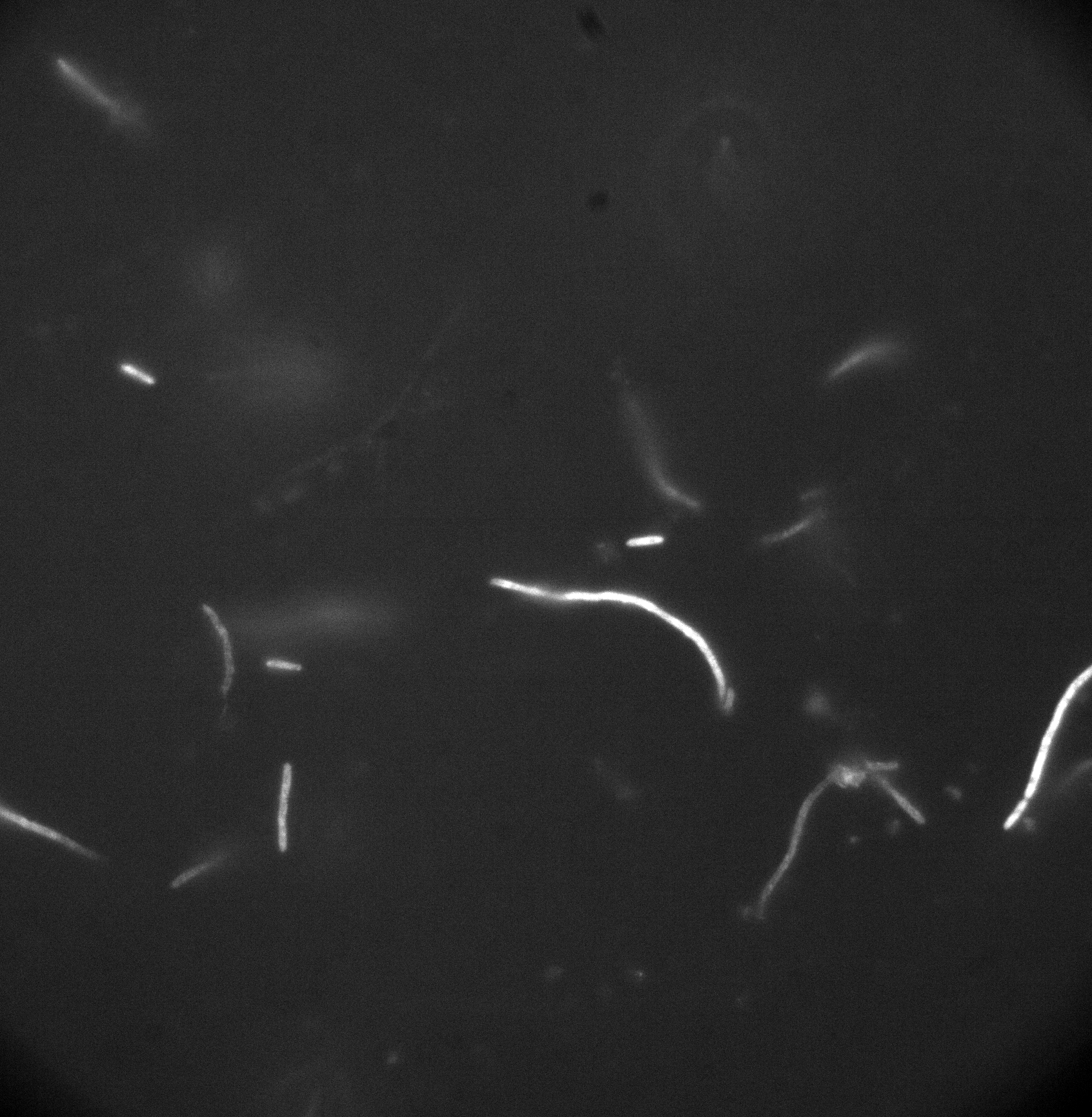

Supplement: Supplementary file 6 — Source data Fig. 3 [file 44321_2025_347_MOESM6_ESM.zip › SD for Fig 3/3G/Fig 3G - middle left (Control +Cephalexin).tif]

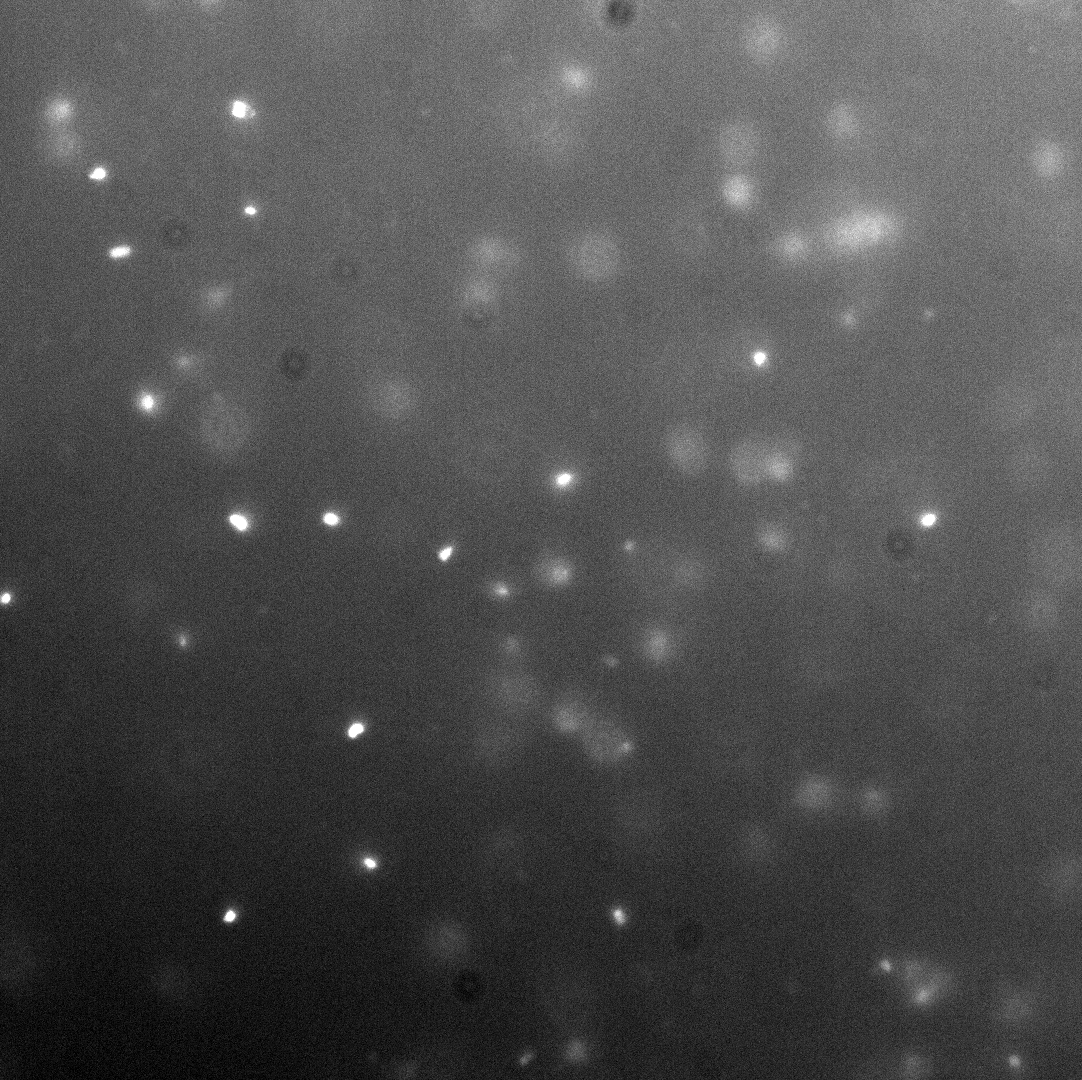

Supplement: Supplementary file 6 — Source data Fig. 3 [file 44321_2025_347_MOESM6_ESM.zip › SD for Fig 3/3G/Fig 3G - left (Untreated control).tif]

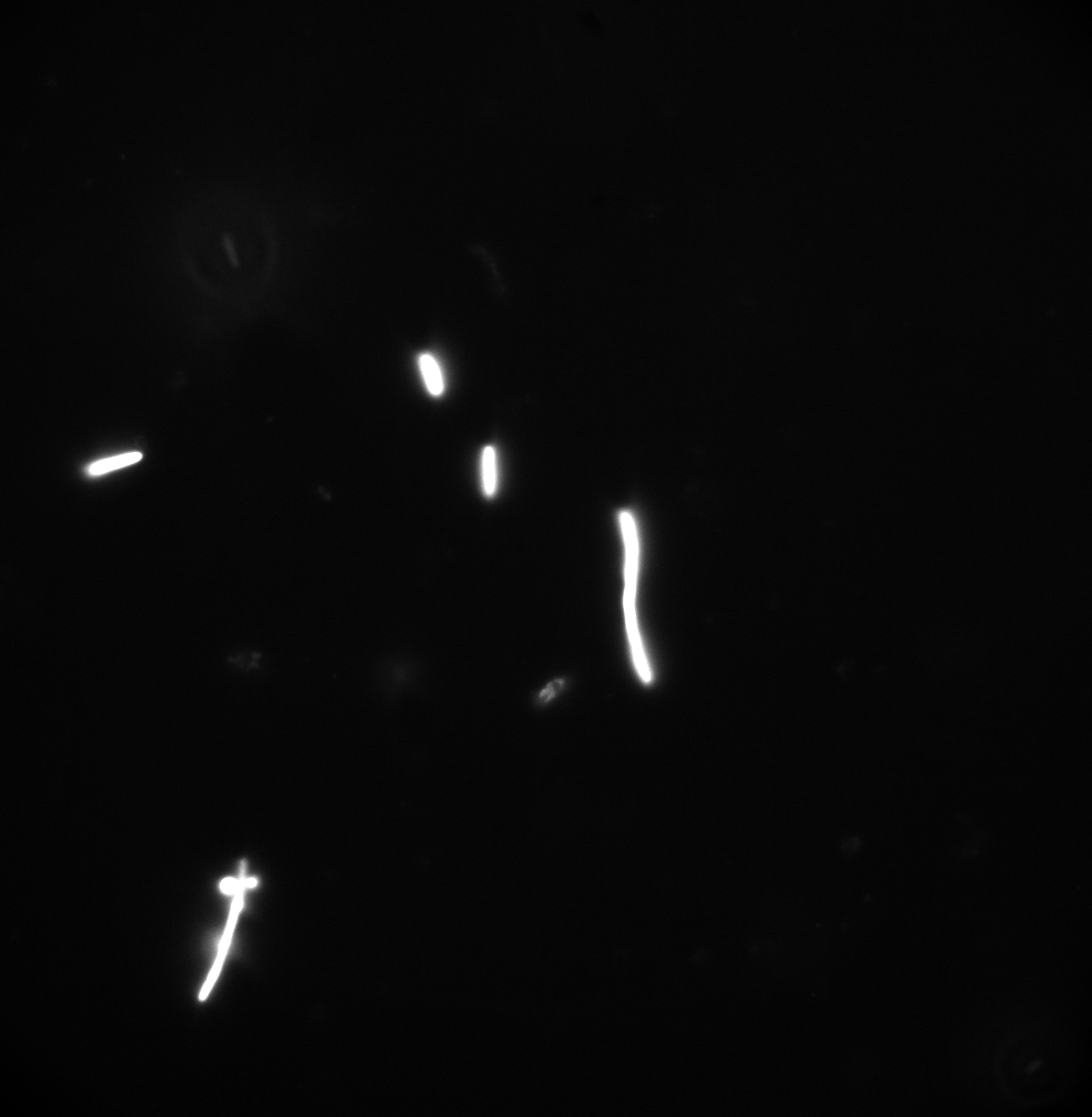

Supplement: Supplementary file 6 — Source data Fig. 3 [file 44321_2025_347_MOESM6_ESM.zip › SD for Fig 3/3G/Fig 3G - right (LYZ:LFN +Cephalexin).tif]

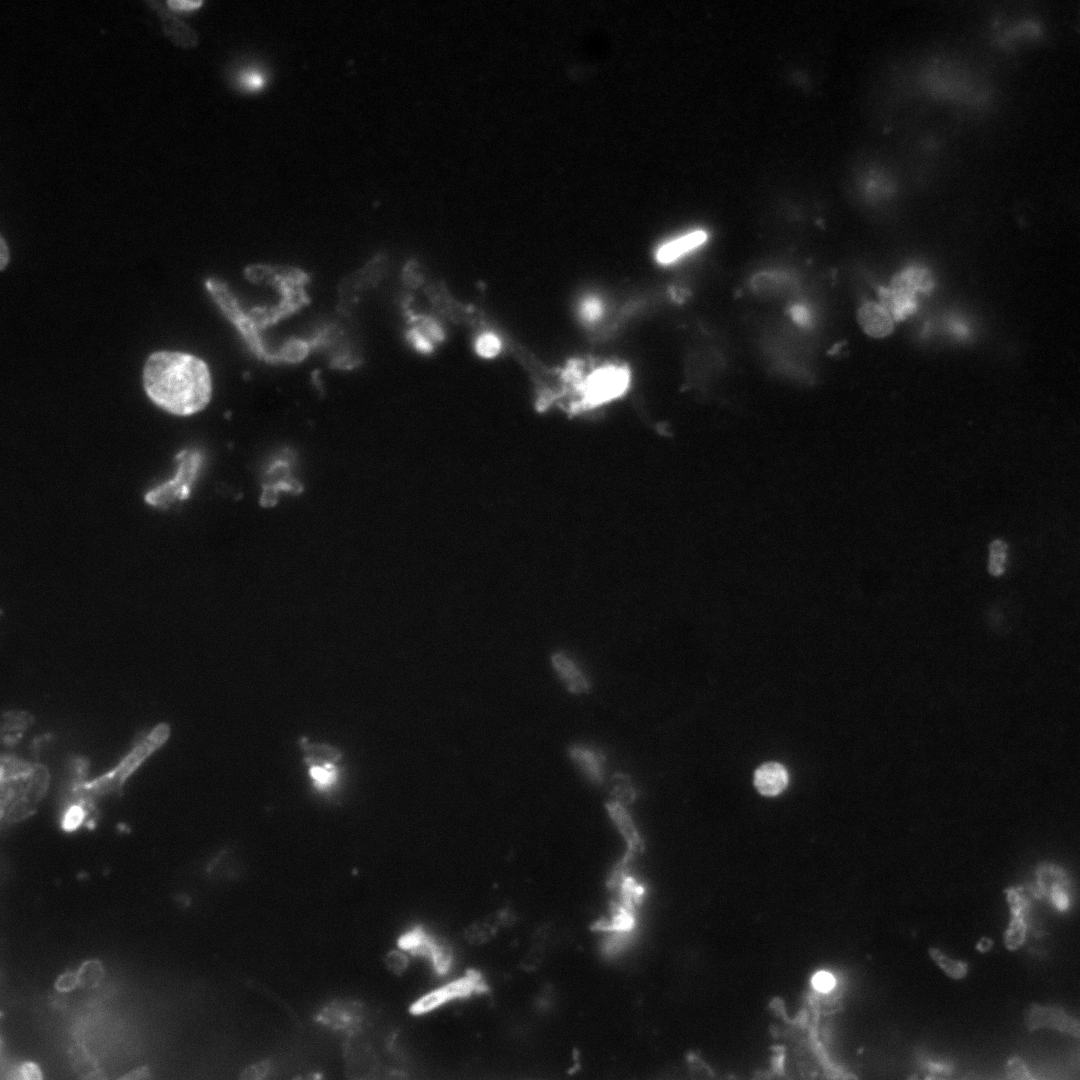

Supplement: Supplementary file 6 — Source data Fig. 3 [file 44321_2025_347_MOESM6_ESM.zip › SD for Fig 3/3G/Fig 3G - middle right (LYZ +Cephalexin).tif]

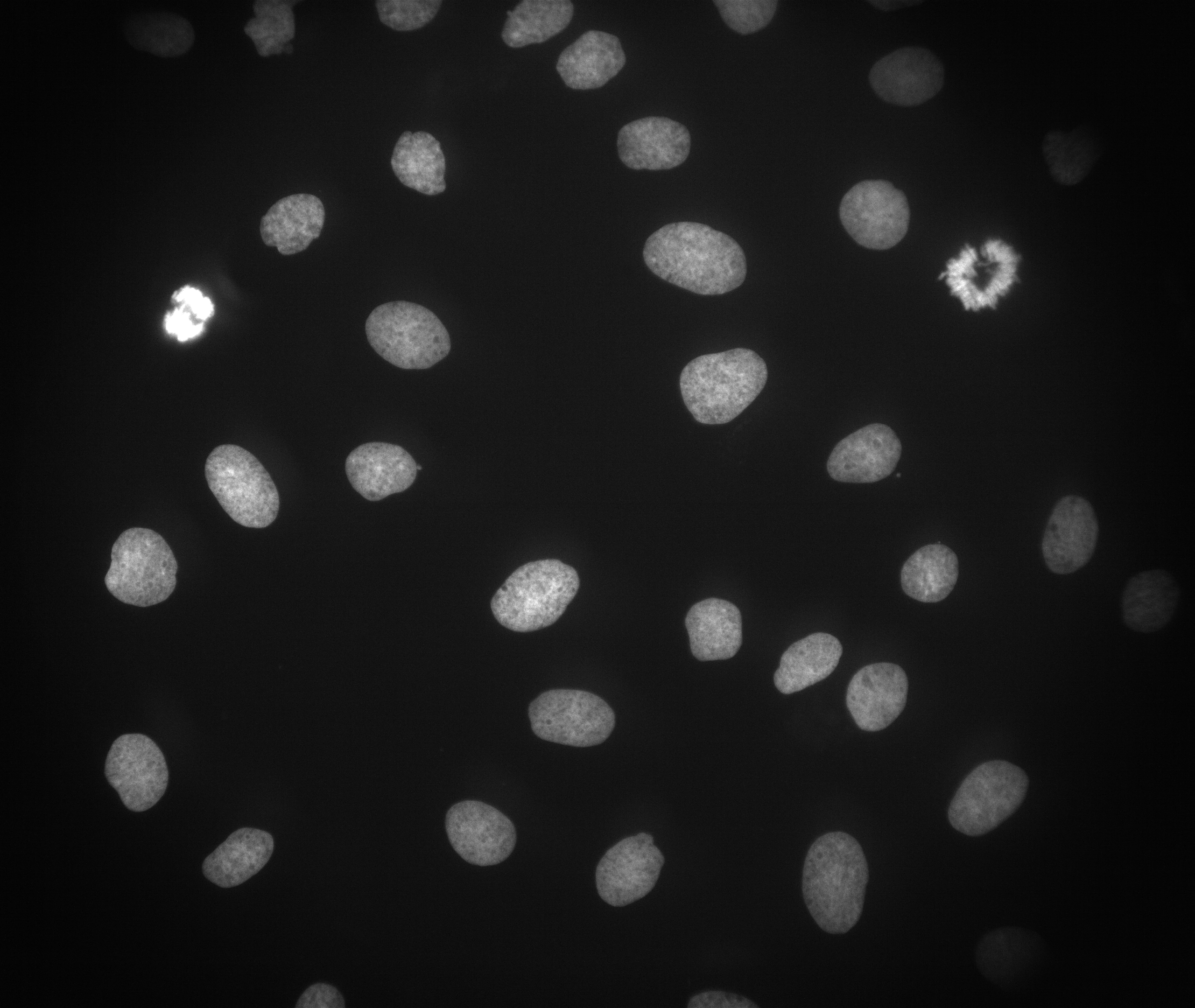

Supplement: Supplementary file 6 — Source data Fig. 3 [file 44321_2025_347_MOESM6_ESM.zip › SD for Fig 3/3A/Fig 3A (left panel)/Fig 3A (left panel).tif]

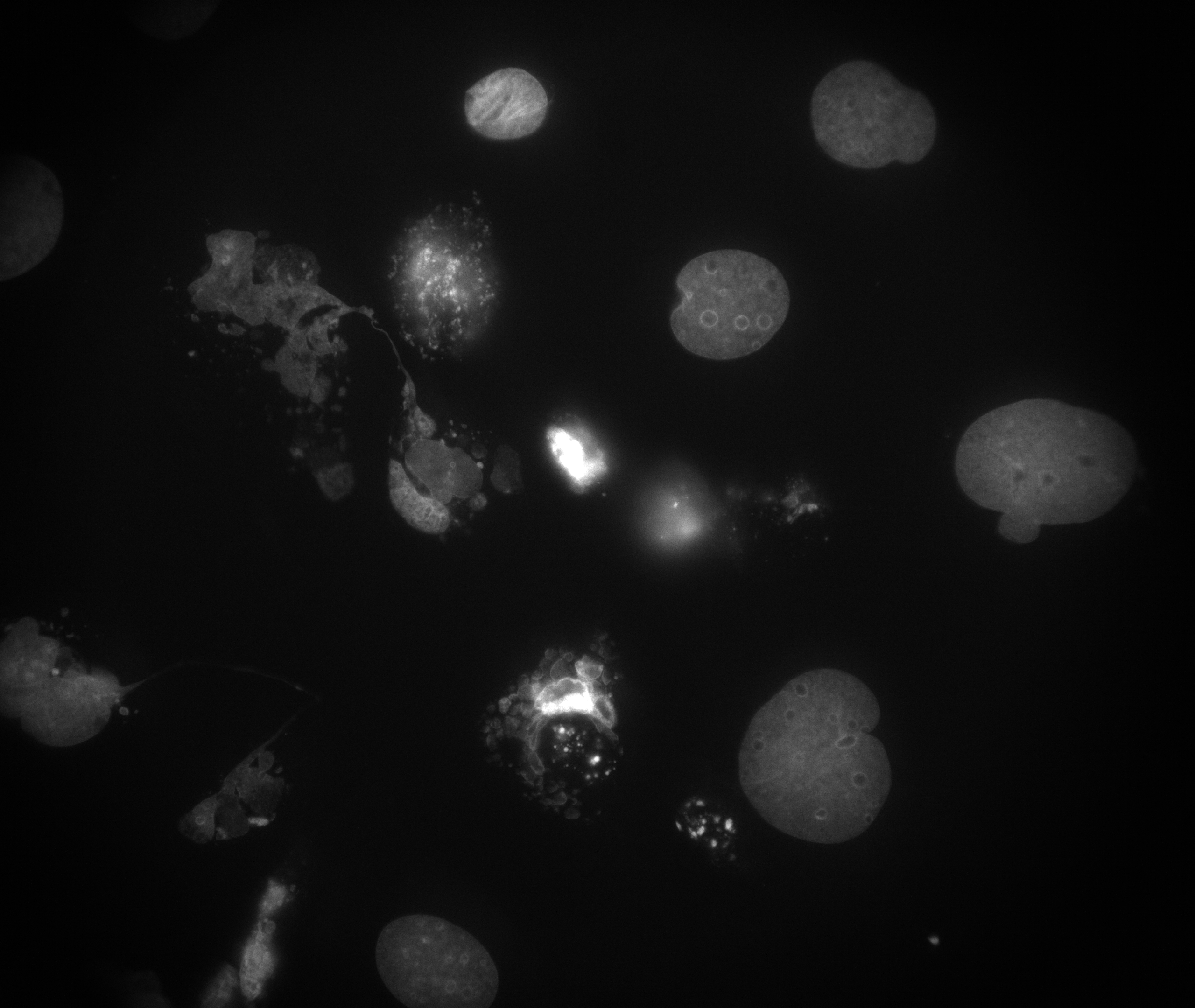

Supplement: Supplementary file 6 — Source data Fig. 3 [file 44321_2025_347_MOESM6_ESM.zip › SD for Fig 3/3A/Fig 3A (middle panel)/Fig 3A (middle panel).tif]

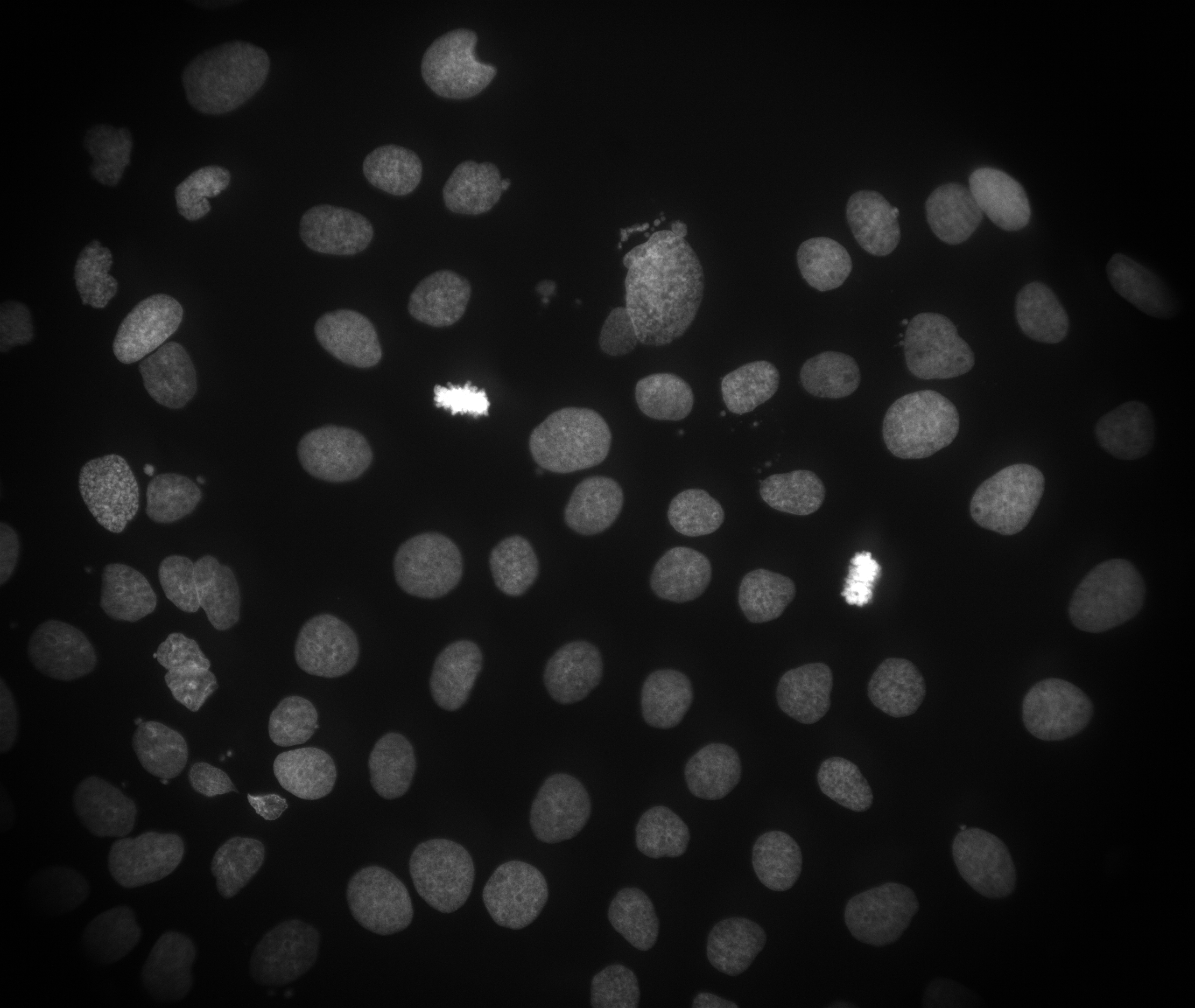

Supplement: Supplementary file 6 — Source data Fig. 3 [file 44321_2025_347_MOESM6_ESM.zip › SD for Fig 3/3A/Fig 3A (right panel)/Fig 3A (right panel).tif]

Fig 5E

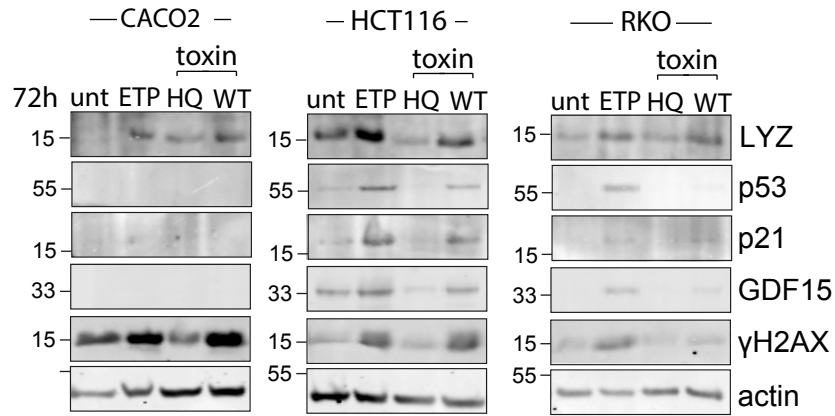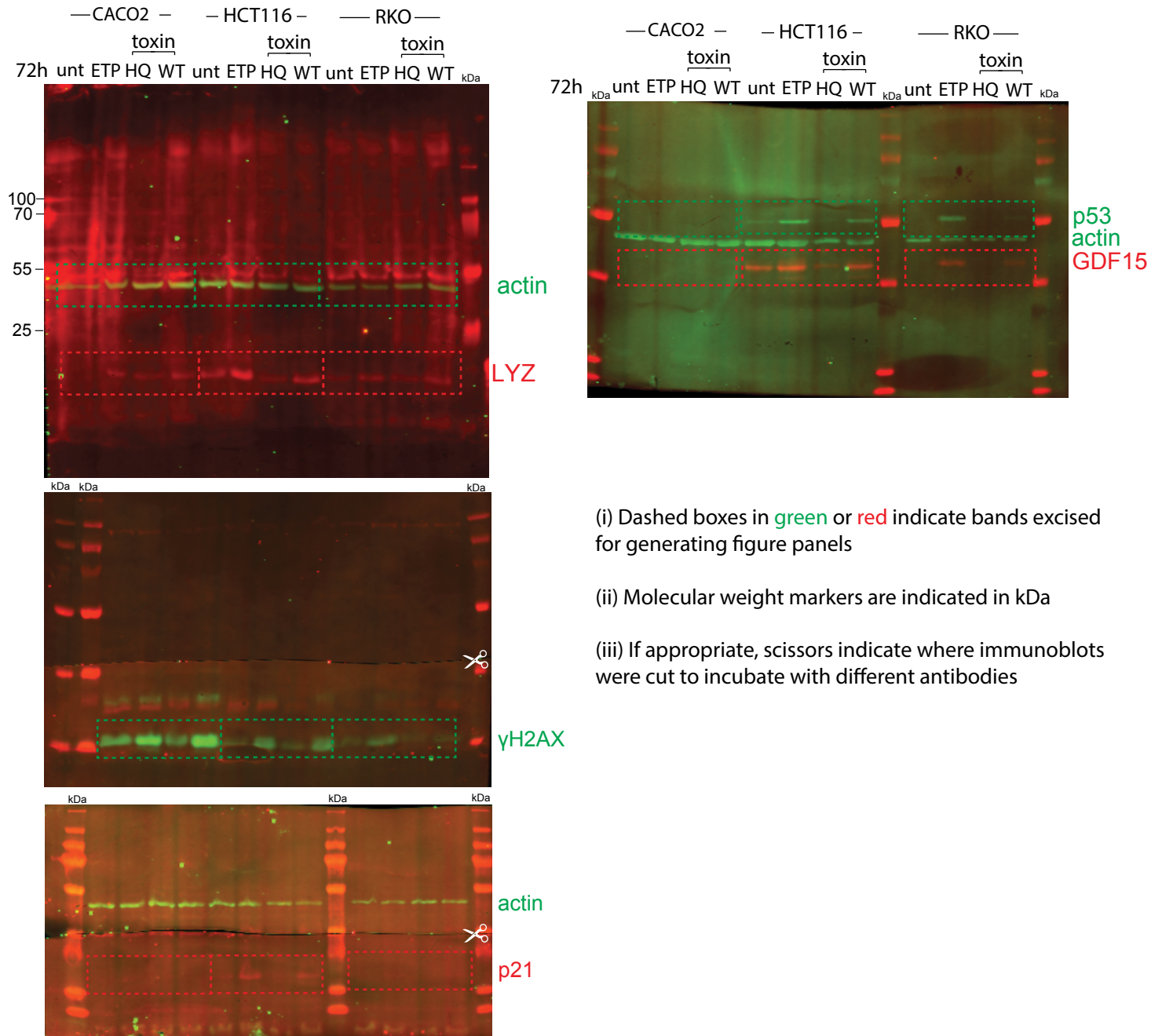

Supplement: Supplementary file 8 — Source data Fig. 5 [file 44321_2025_347_MOESM8_ESM.zip › SD for Fig 5/5╬ò/5╬ò_SD.pdf]

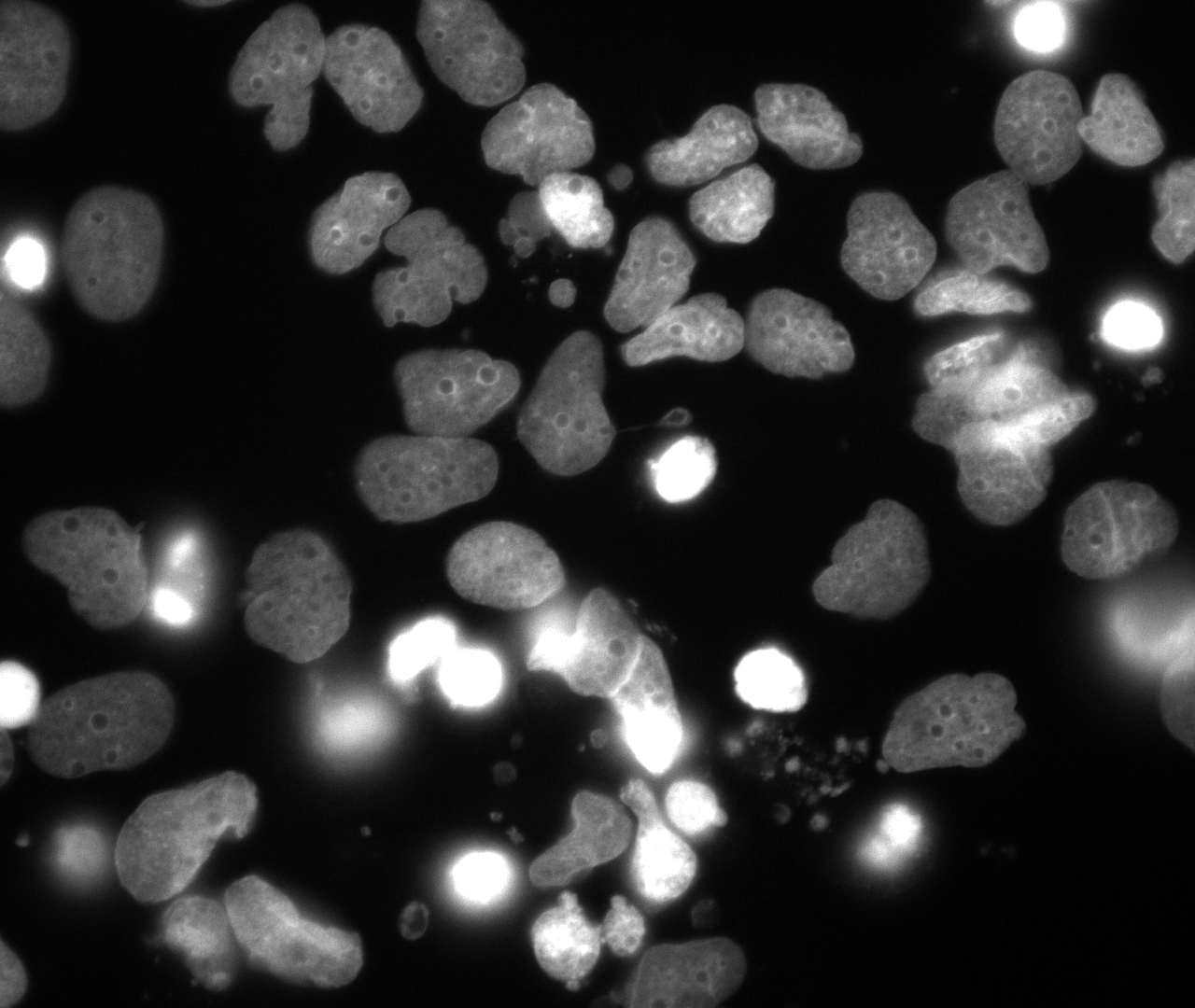

Supplement: Supplementary file 8 — Source data Fig. 5 [file 44321_2025_347_MOESM8_ESM.zip › SD for Fig 5/5A/5A (Bottom Panel - ETP)/5A (ETP + Caffeine).tif]

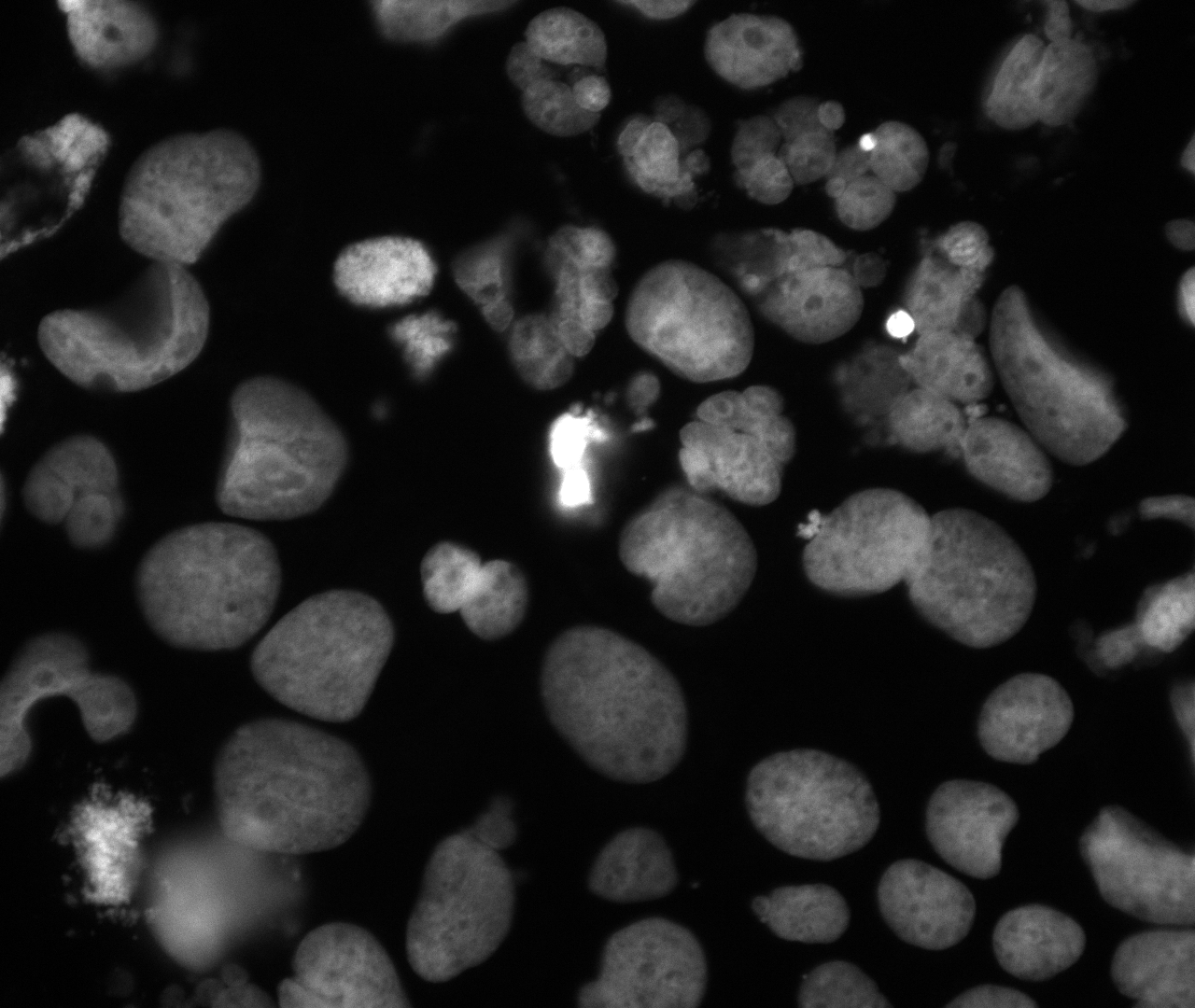

Supplement: Supplementary file 8 — Source data Fig. 5 [file 44321_2025_347_MOESM8_ESM.zip › SD for Fig 5/5A/5A (Bottom Panel - ETP)/5A (ETP - Caffeine).tif]

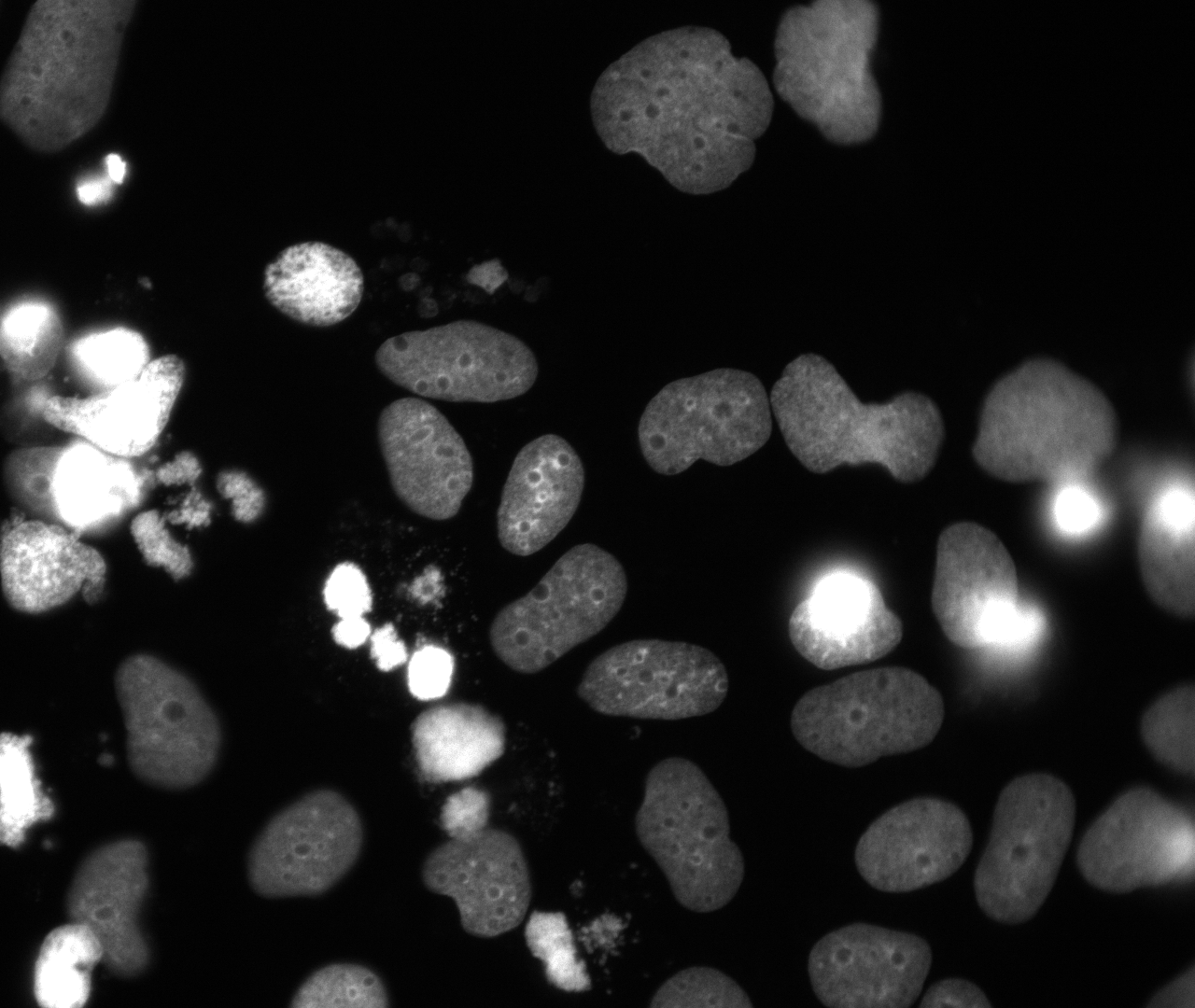

Supplement: Supplementary file 8 — Source data Fig. 5 [file 44321_2025_347_MOESM8_ESM.zip › SD for Fig 5/5A/5A (middle Panel - TxWT) /5A (TxWT + Caffeine).tif]

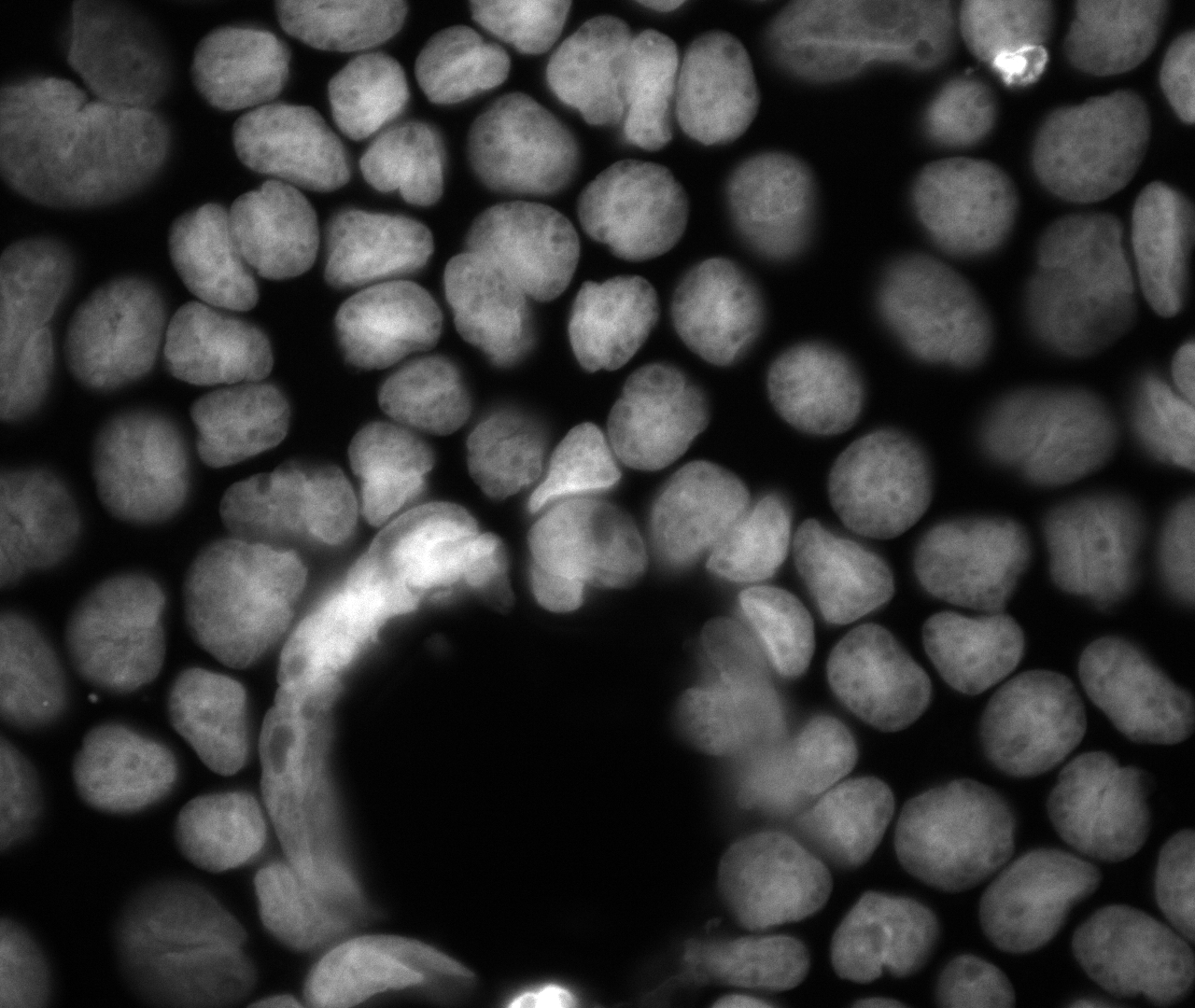

Supplement: Supplementary file 8 — Source data Fig. 5 [file 44321_2025_347_MOESM8_ESM.zip › SD for Fig 5/5A/5A (middle Panel - TxWT) /5A (TxWT - Caffeine).tif]

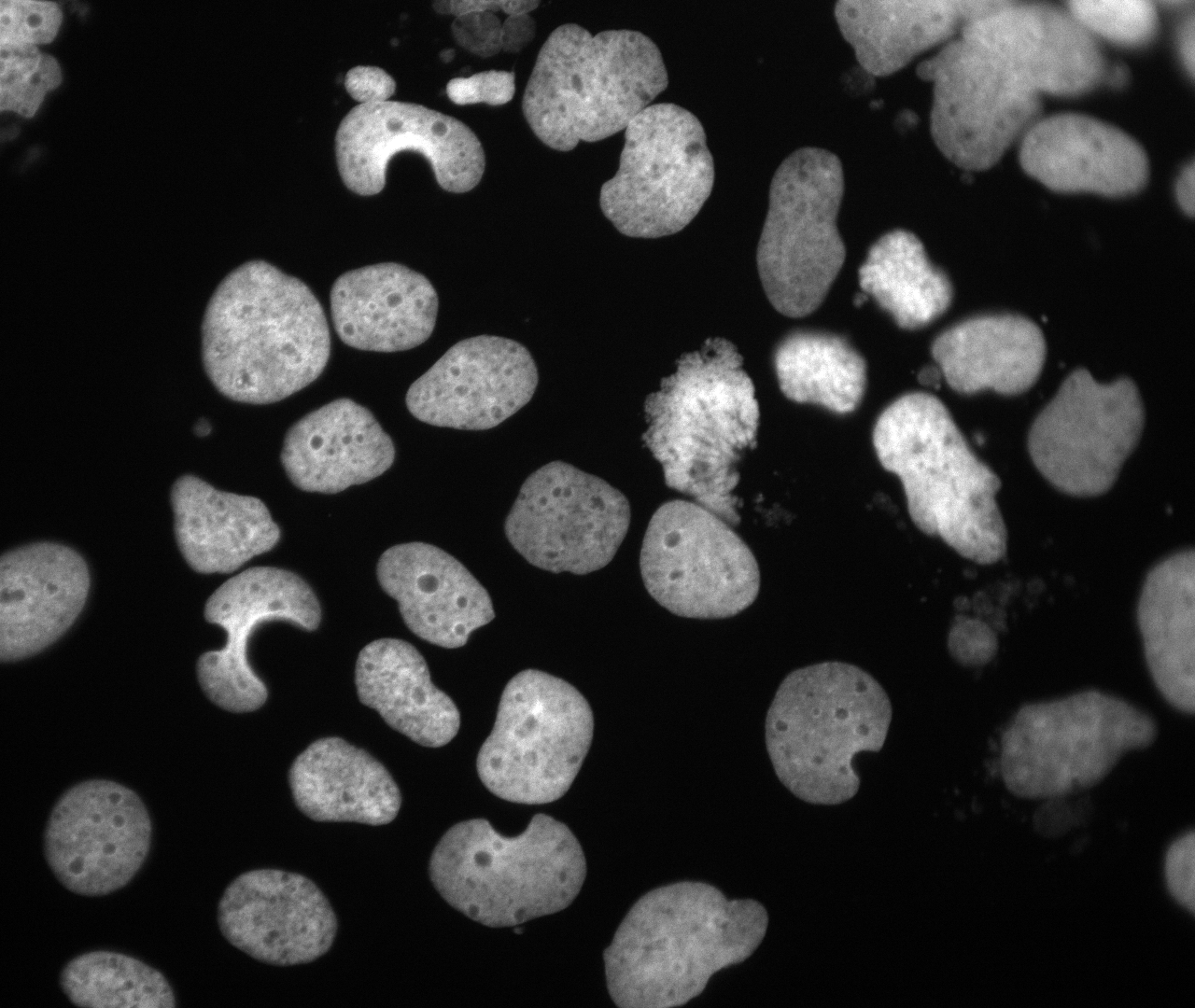

Supplement: Supplementary file 8 — Source data Fig. 5 [file 44321_2025_347_MOESM8_ESM.zip › SD for Fig 5/5A/5A (Top Panel - unt)/5A (Unt + Caffeine).tif]

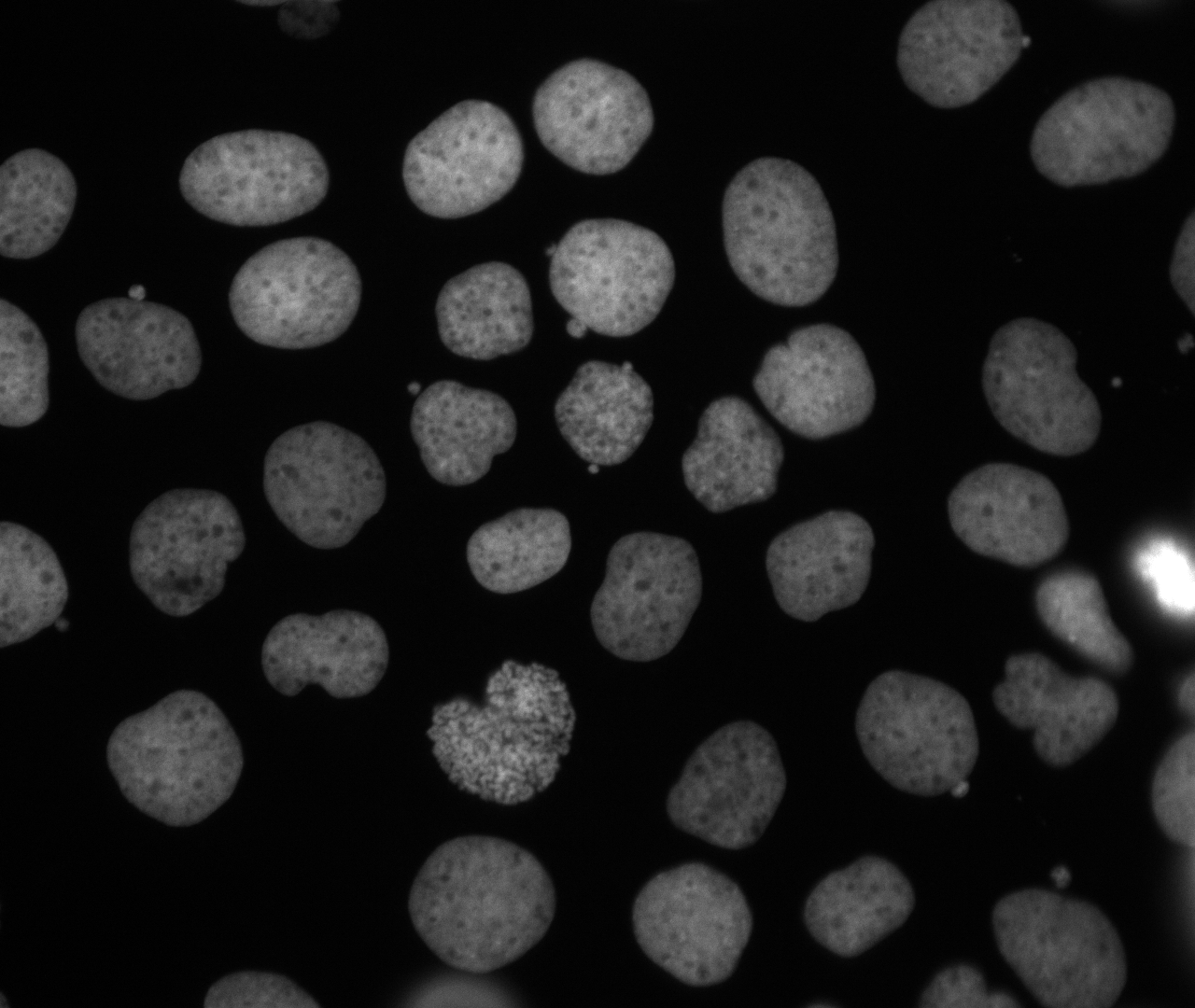

Supplement: Supplementary file 8 — Source data Fig. 5 [file 44321_2025_347_MOESM8_ESM.zip › SD for Fig 5/5A/5A (Top Panel - unt)/5A (Unt - Caffeine).tif]

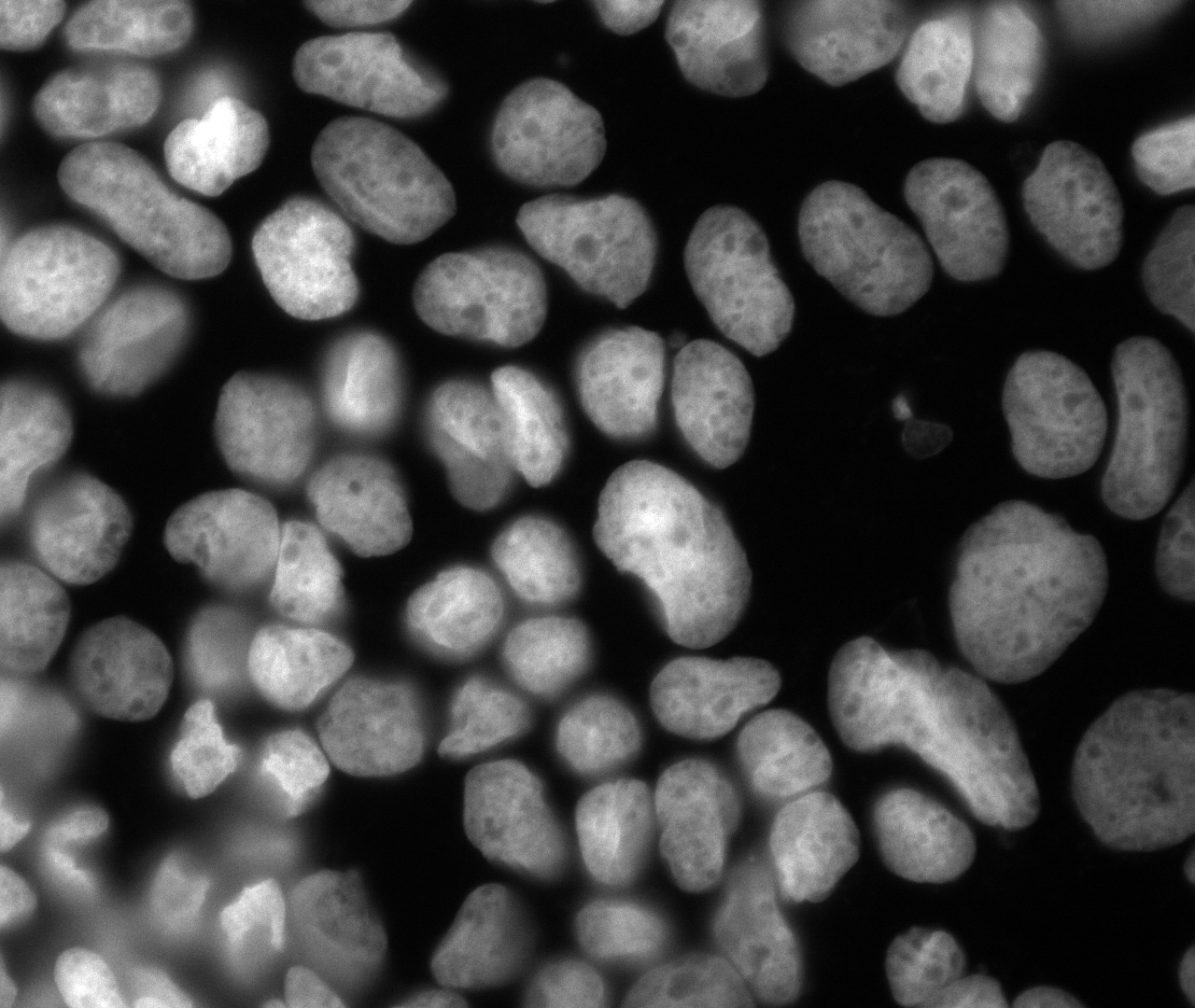

Supplement: Supplementary file 8 — Source data Fig. 5 [file 44321_2025_347_MOESM8_ESM.zip › SD for Fig 5/5B/5B (middle panel - TxWT)/5B (Tx-WT - Caffeine).tif]

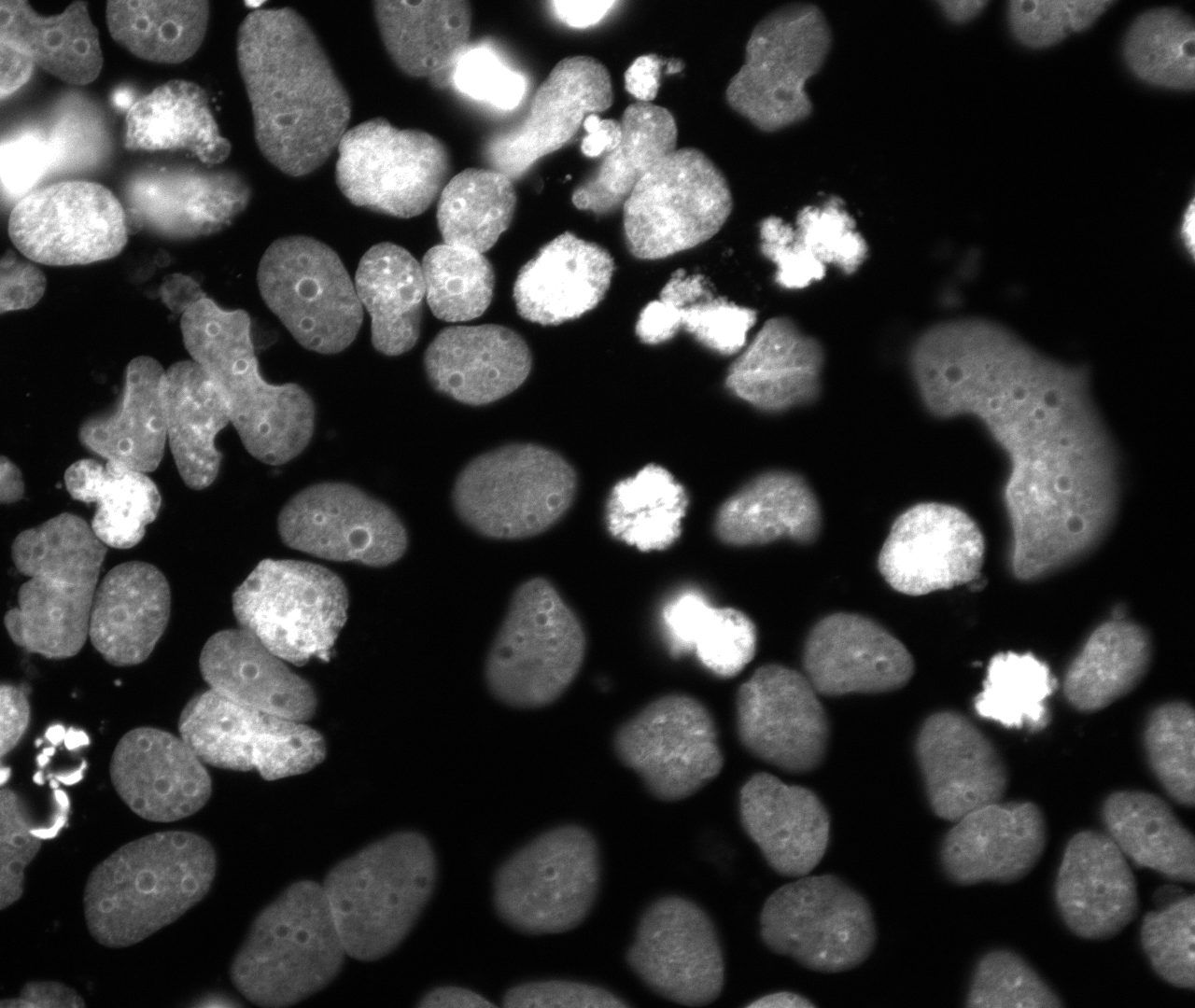

Supplement: Supplementary file 8 — Source data Fig. 5 [file 44321_2025_347_MOESM8_ESM.zip › SD for Fig 5/5B/5B (middle panel - TxWT)/5B (Tx-WT + Caffeine).tif]

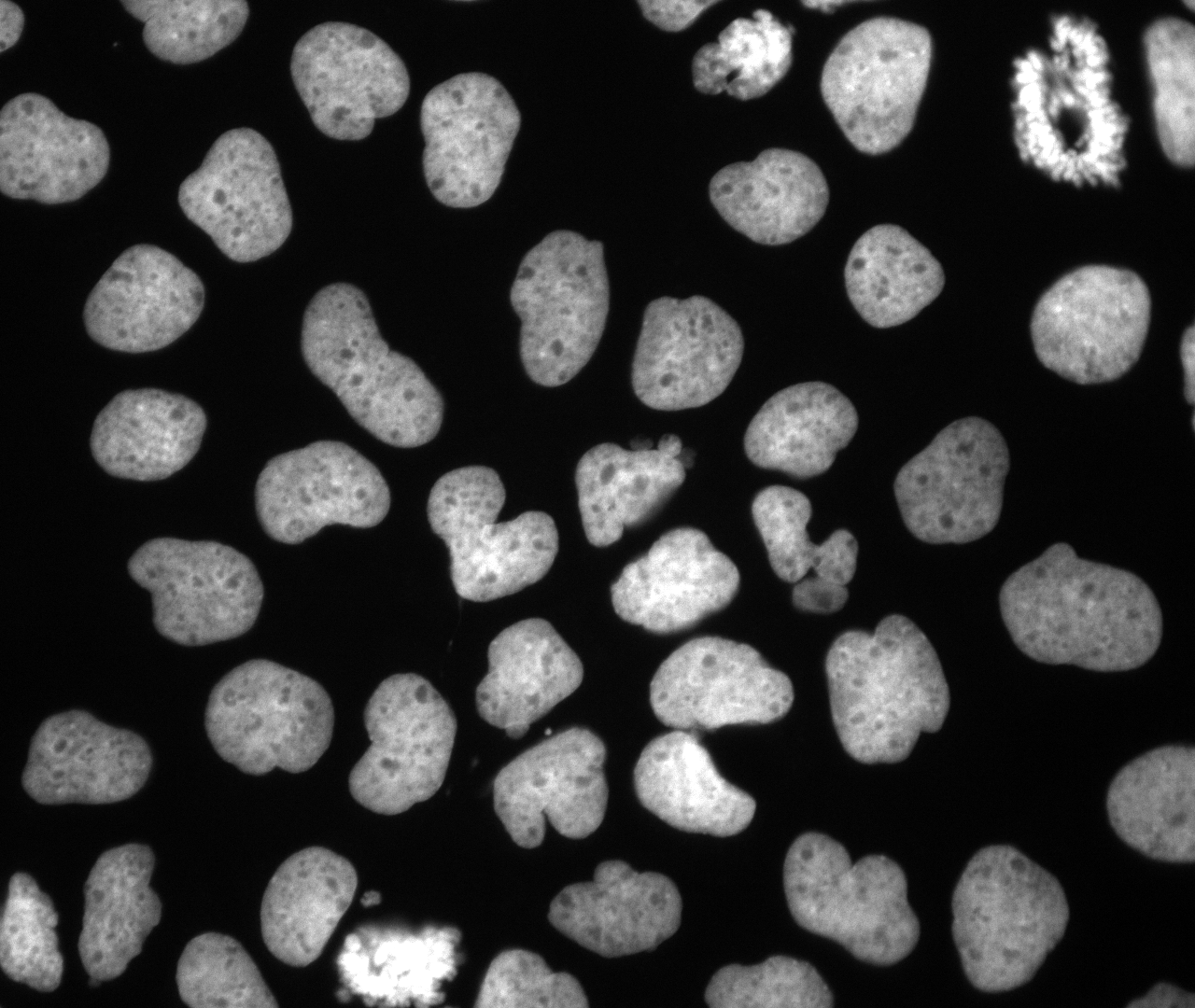

Supplement: Supplementary file 8 — Source data Fig. 5 [file 44321_2025_347_MOESM8_ESM.zip › SD for Fig 5/5B/5B (Top Panel - unt)/5B (Unt - Caffeine).tif]

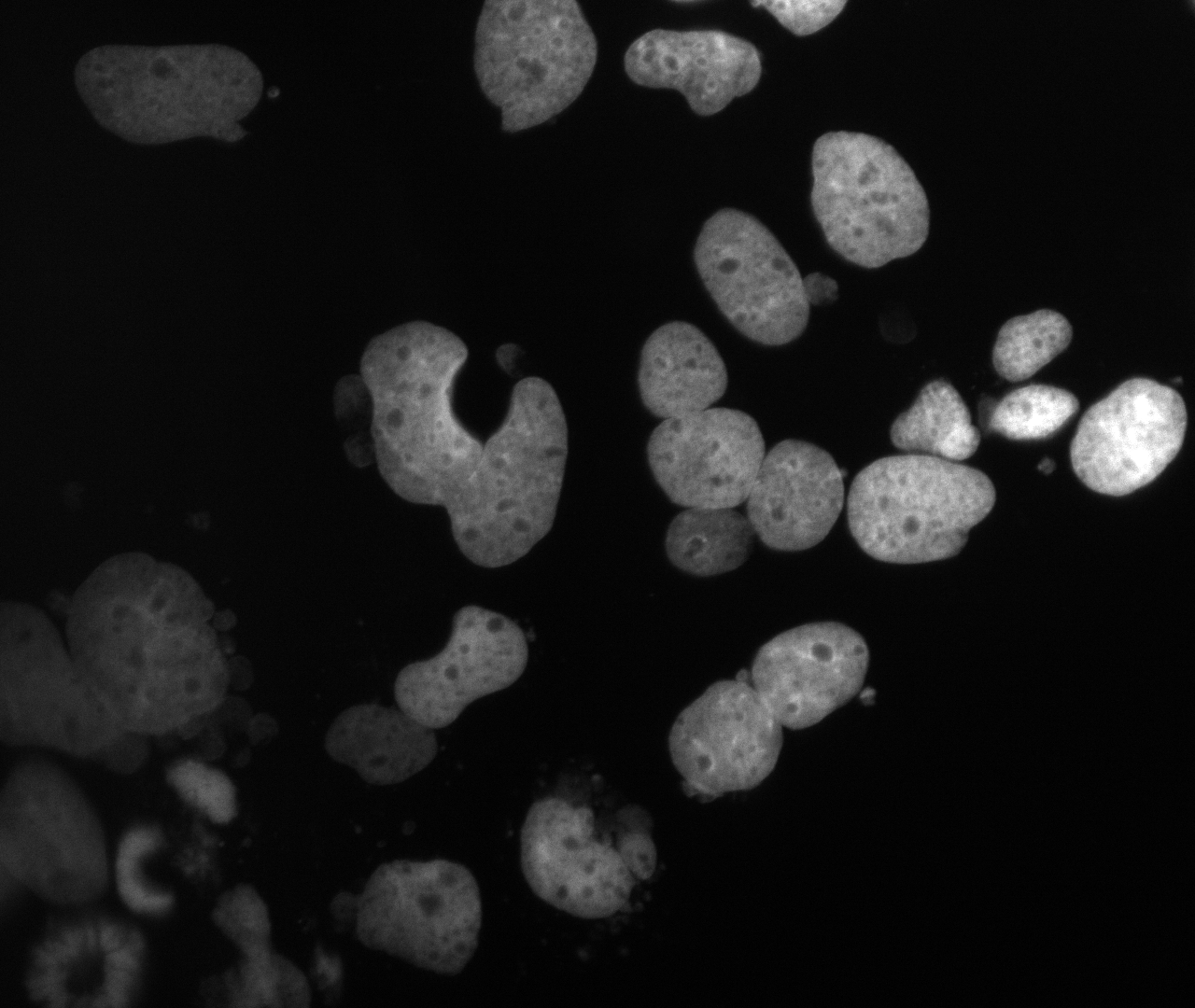

Supplement: Supplementary file 8 — Source data Fig. 5 [file 44321_2025_347_MOESM8_ESM.zip › SD for Fig 5/5B/5B (Top Panel - unt)/5B (Unt + Caffeine).tif]

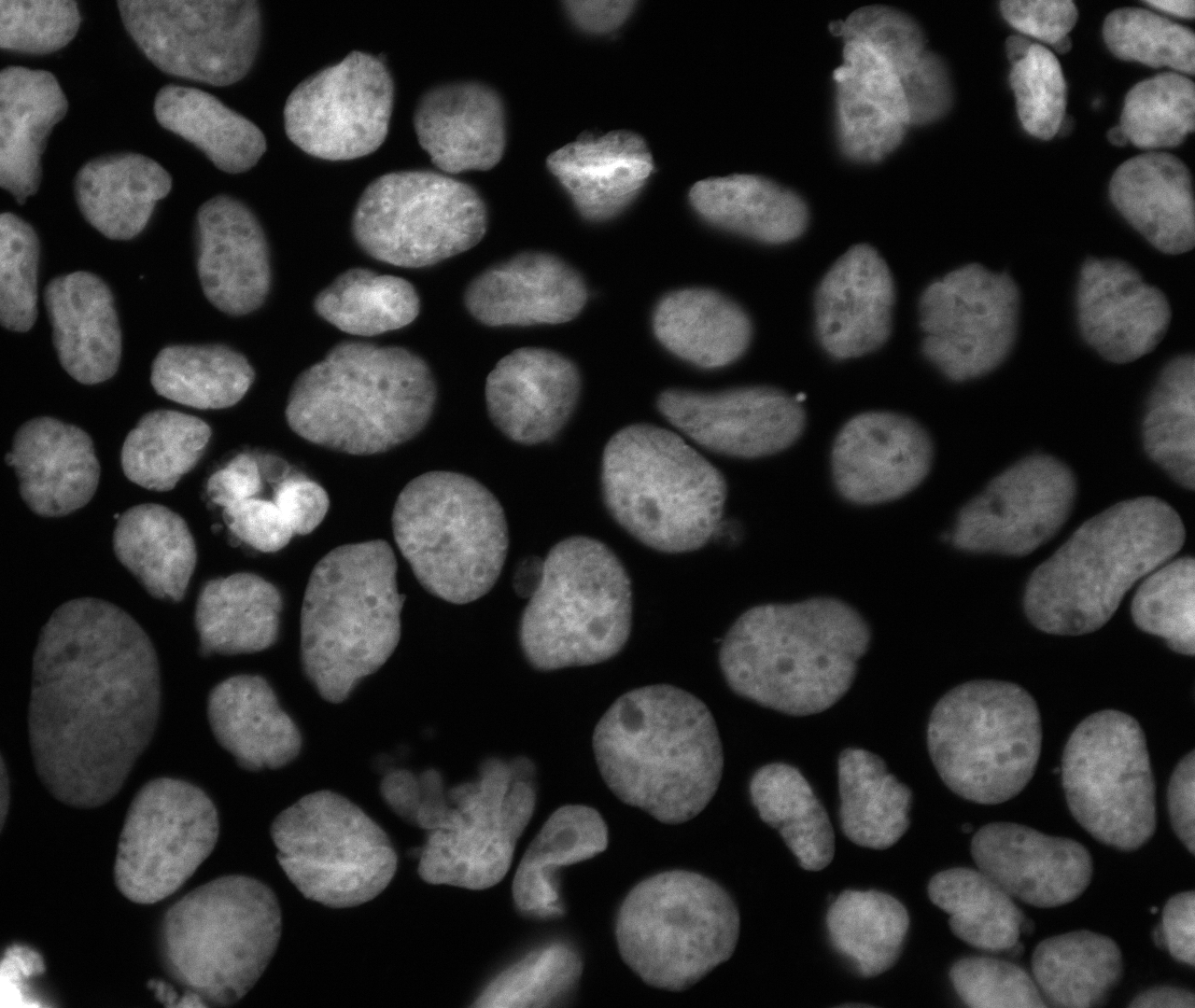

Supplement: Supplementary file 8 — Source data Fig. 5 [file 44321_2025_347_MOESM8_ESM.zip › SD for Fig 5/5B/5B (Bottom Panel - ETP)/5B (ETP - Caffeine).tif]

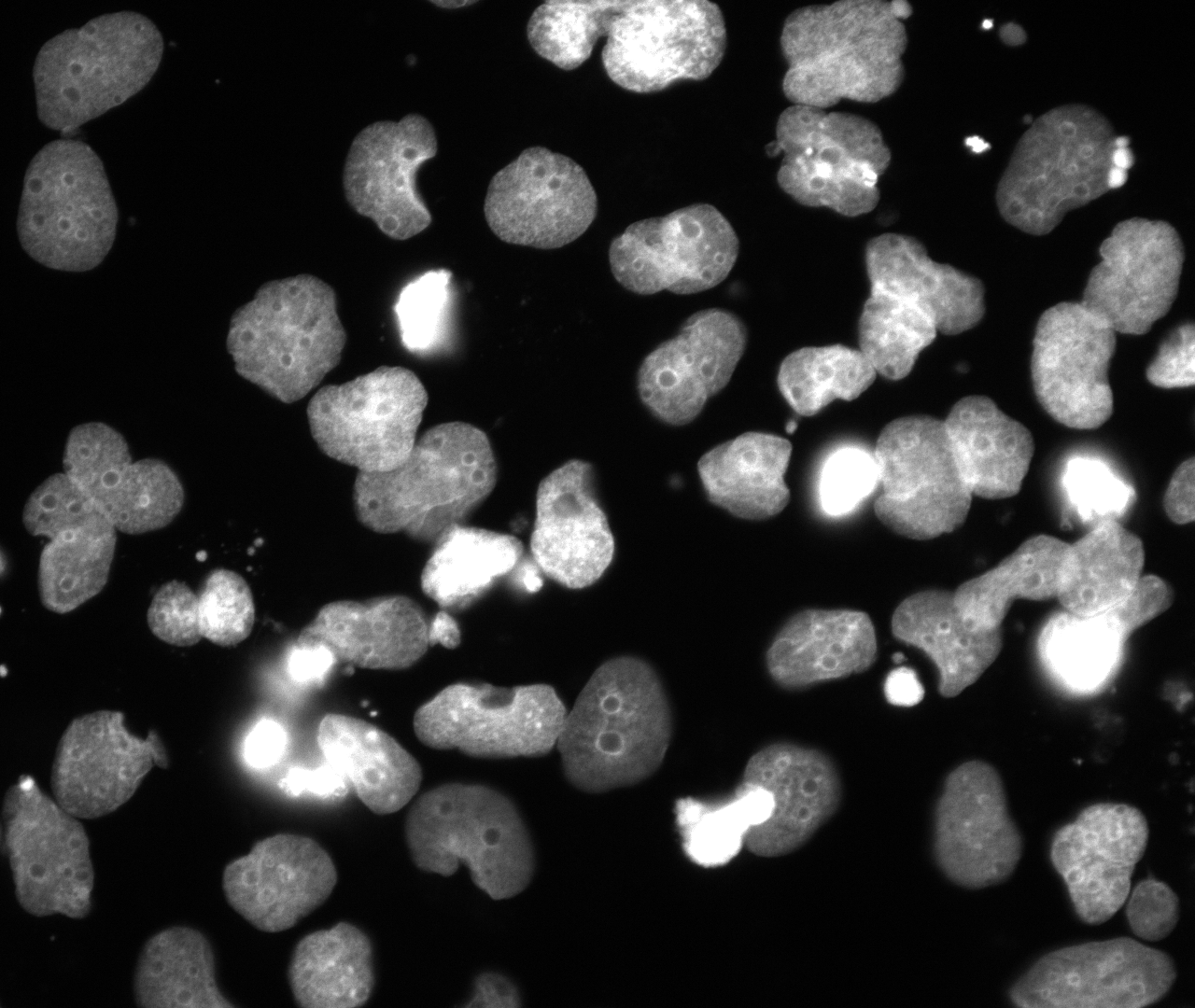

Supplement: Supplementary file 8 — Source data Fig. 5 [file 44321_2025_347_MOESM8_ESM.zip › SD for Fig 5/5B/5B (Bottom Panel - ETP)/5B (ETP + Caffeine).tif]

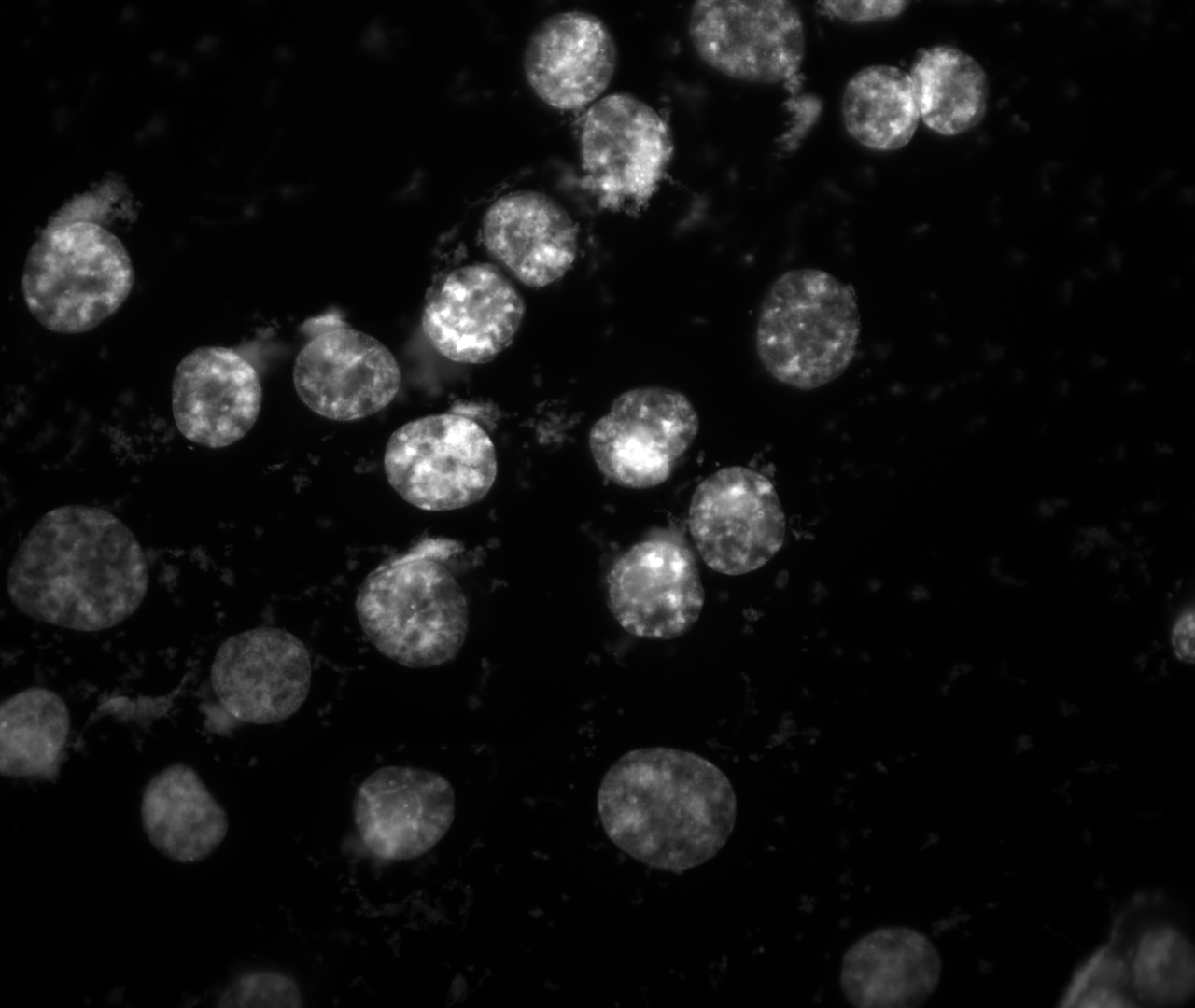

Supplement: Supplementary file 9 — Source data Fig. 6 [file 44321_2025_347_MOESM9_ESM.zip › SD for Fig 6/6A/6A (Top panel Parental).tif]

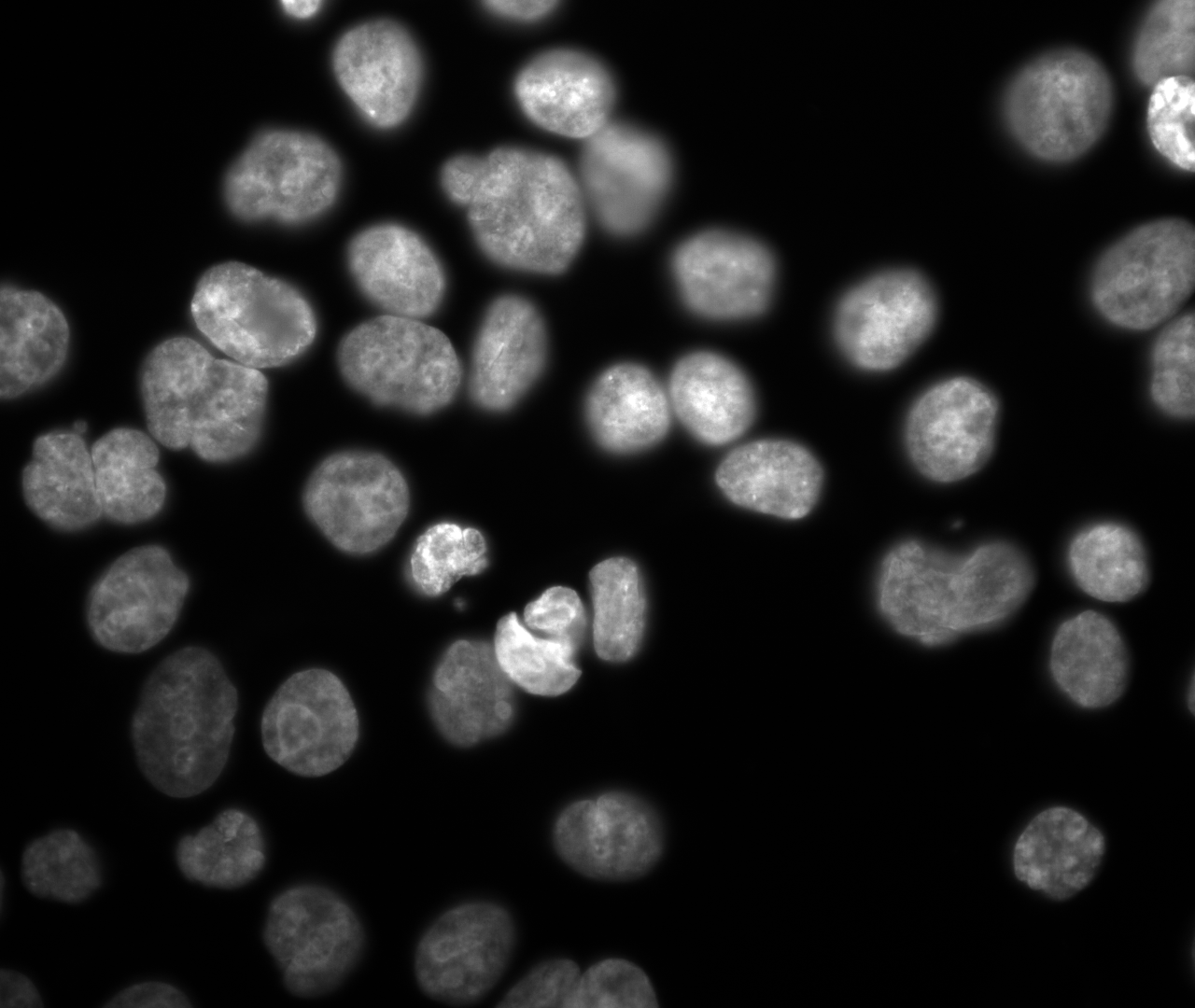

Supplement: Supplementary file 9 — Source data Fig. 6 [file 44321_2025_347_MOESM9_ESM.zip › SD for Fig 6/6A/6A (bottom panel TP53 KO).tif]

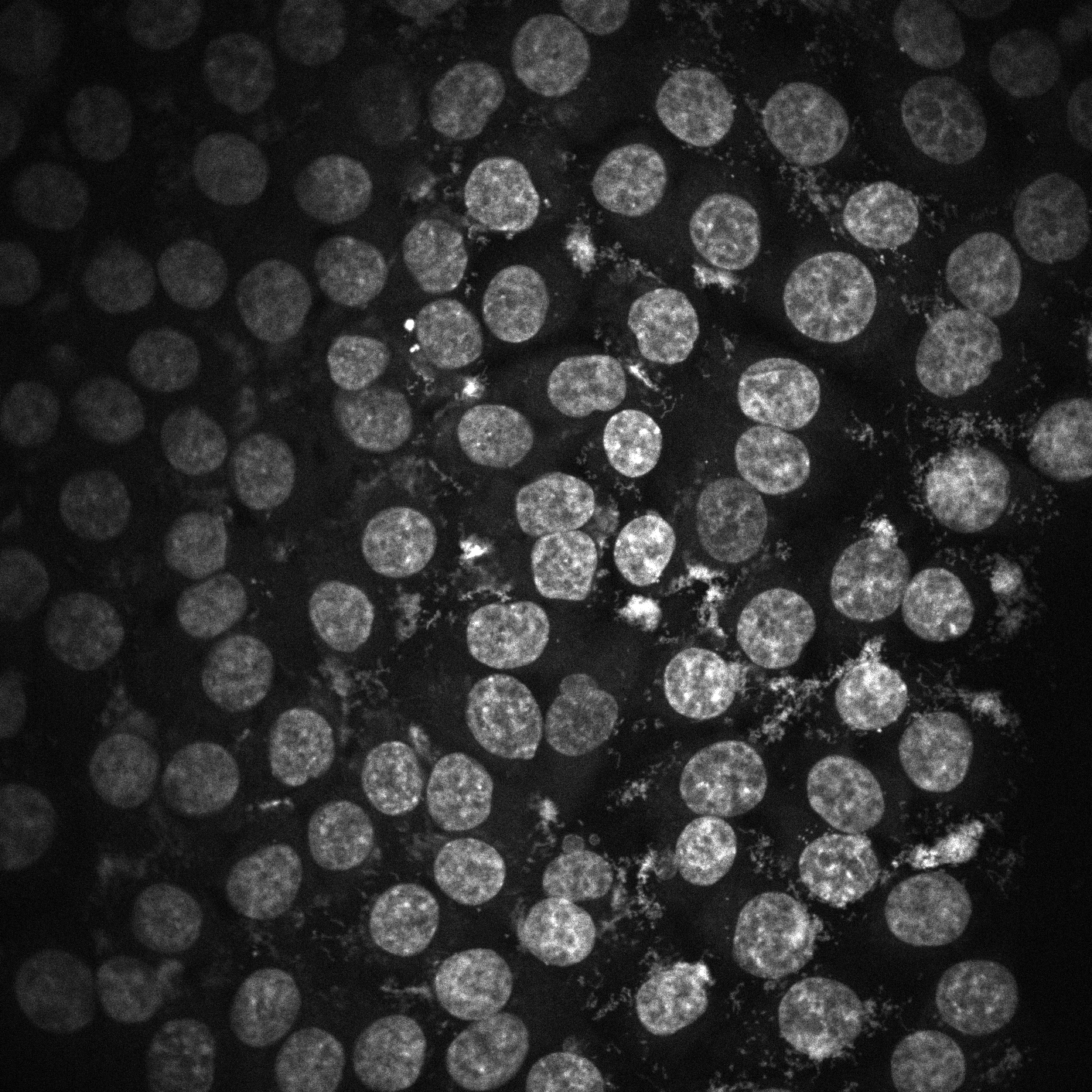

Supplement: Supplementary file 9 — Source data Fig. 6 [file 44321_2025_347_MOESM9_ESM.zip › SD for Fig 6/6H/6H (right panel - exogenous LYZ).tif]

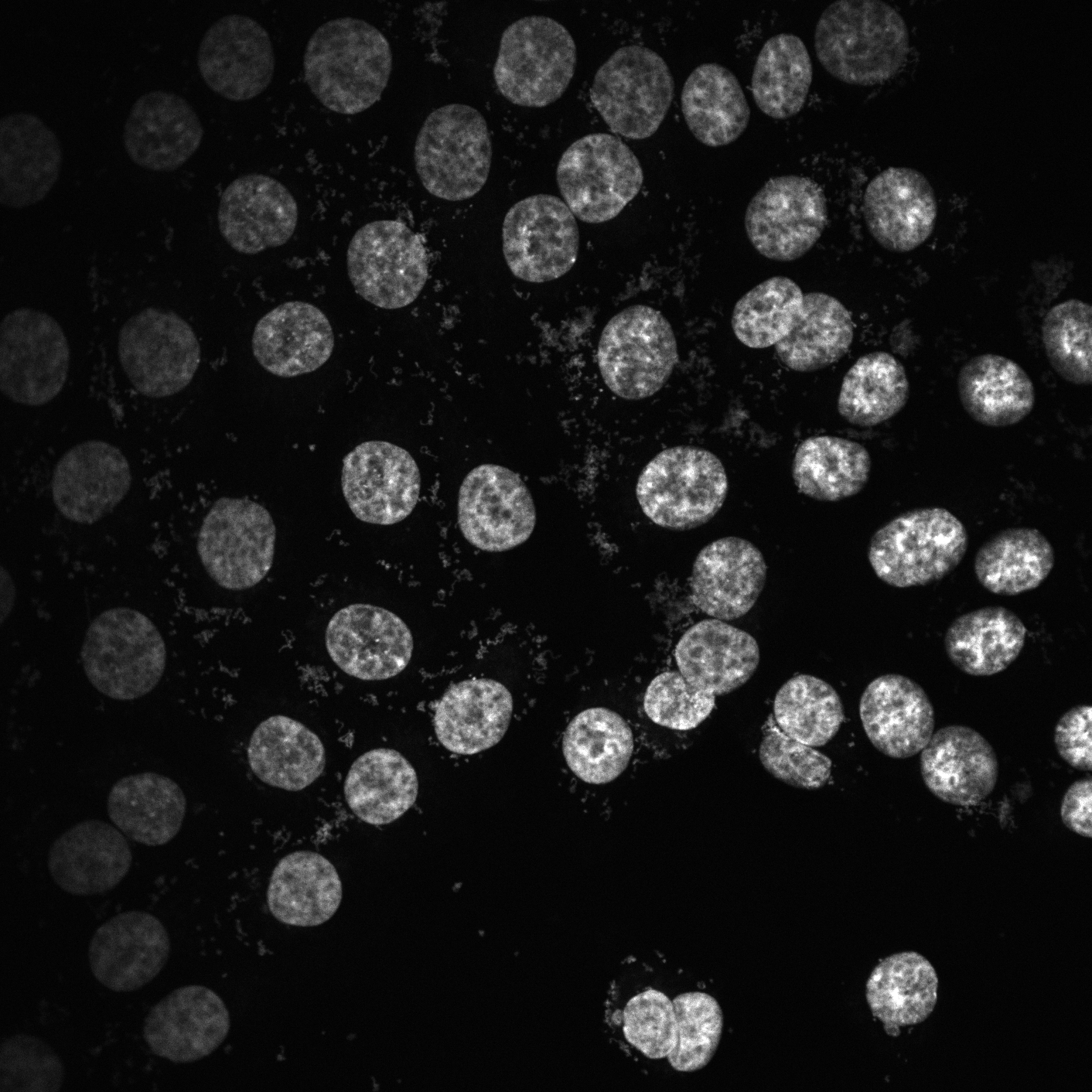

Supplement: Supplementary file 9 — Source data Fig. 6 [file 44321_2025_347_MOESM9_ESM.zip › SD for Fig 6/6H/6H (middle panel - control).tif]

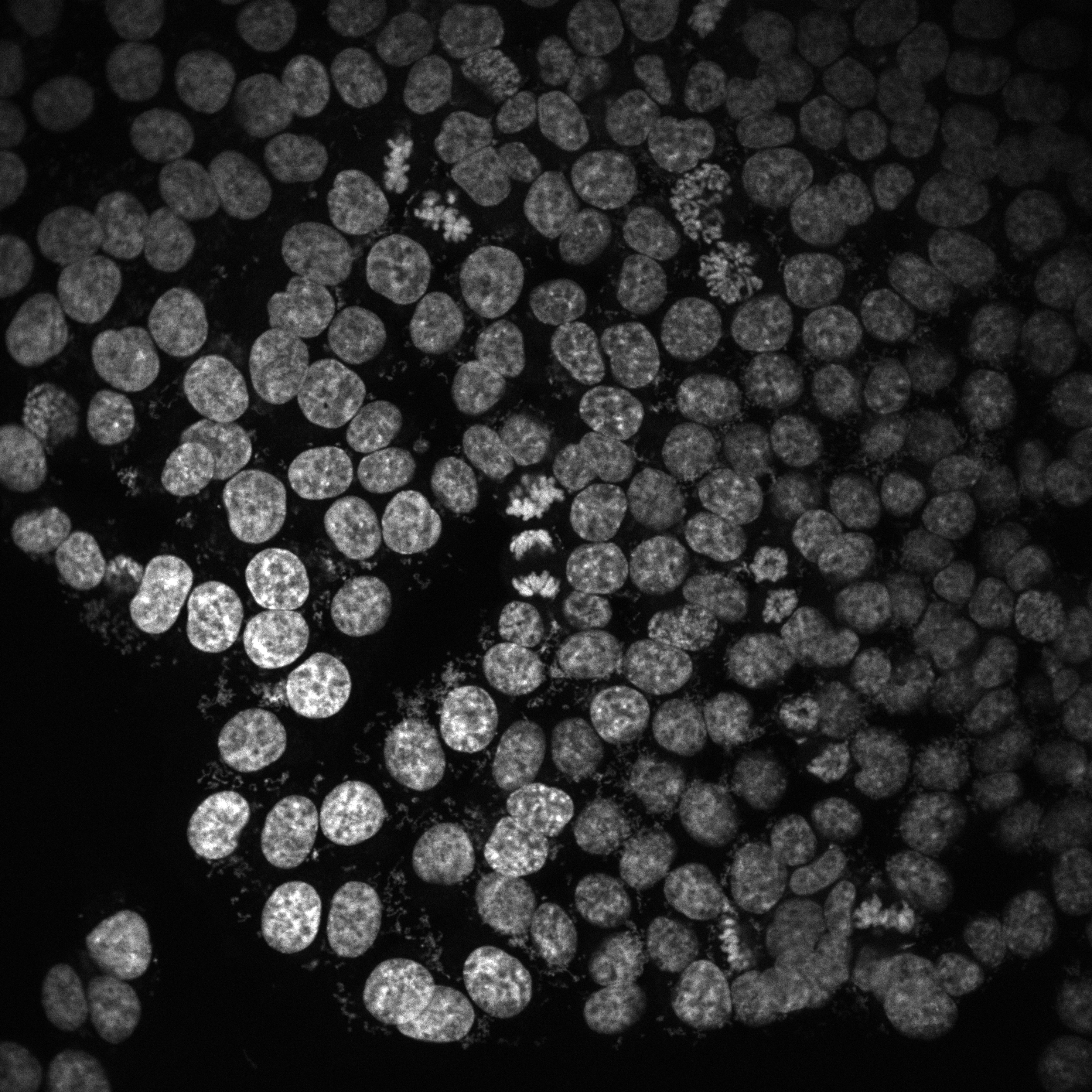

Supplement: Supplementary file 9 — Source data Fig. 6 [file 44321_2025_347_MOESM9_ESM.zip › SD for Fig 6/6H/6H (left panel - Untreated).tif]

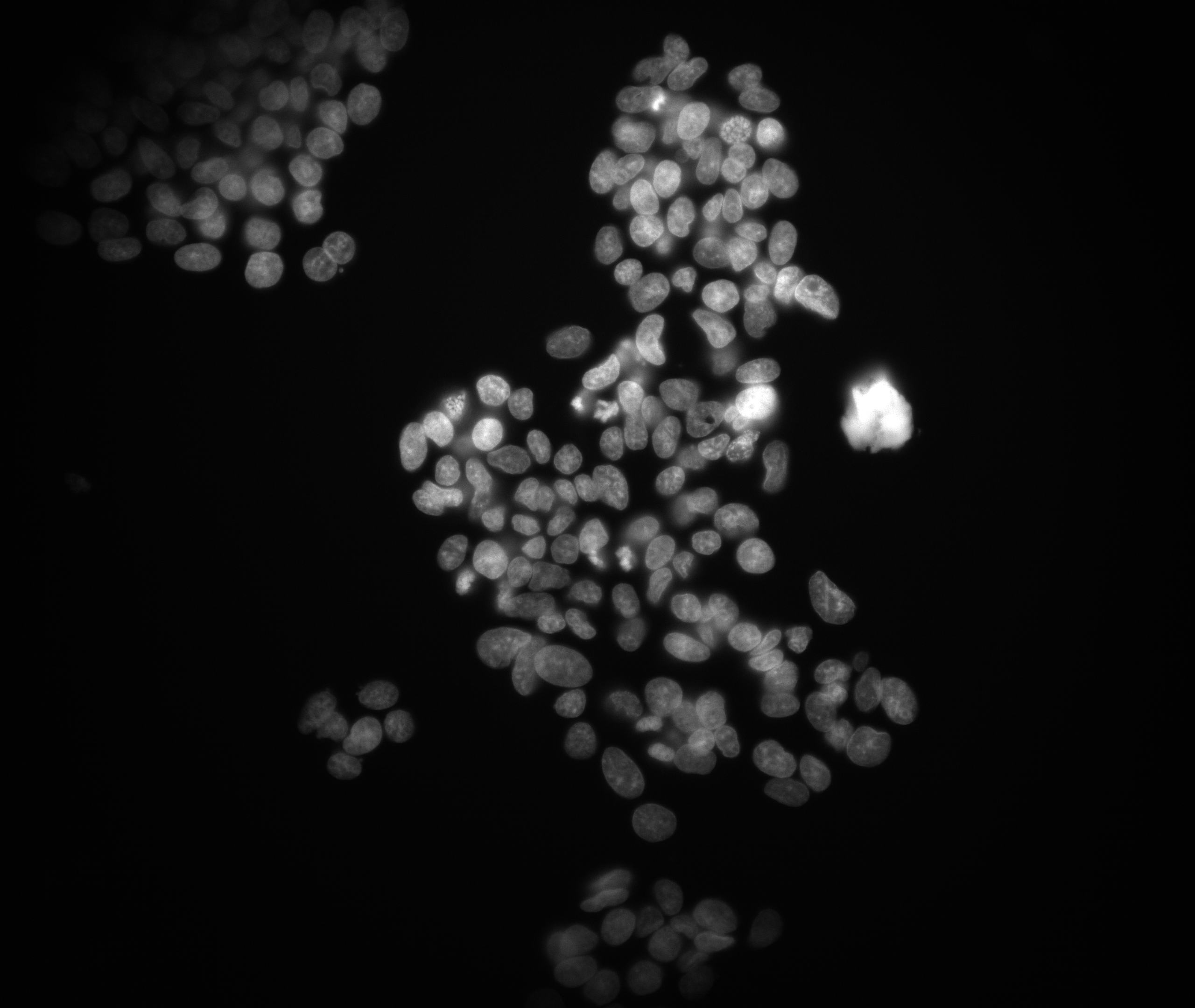

Supplement: Supplementary file 10 — Figure EV1 Source Data [file 44321_2025_347_MOESM10_ESM.zip › SD for Fig EV1/EV1A/EV1A (HQ).tif]

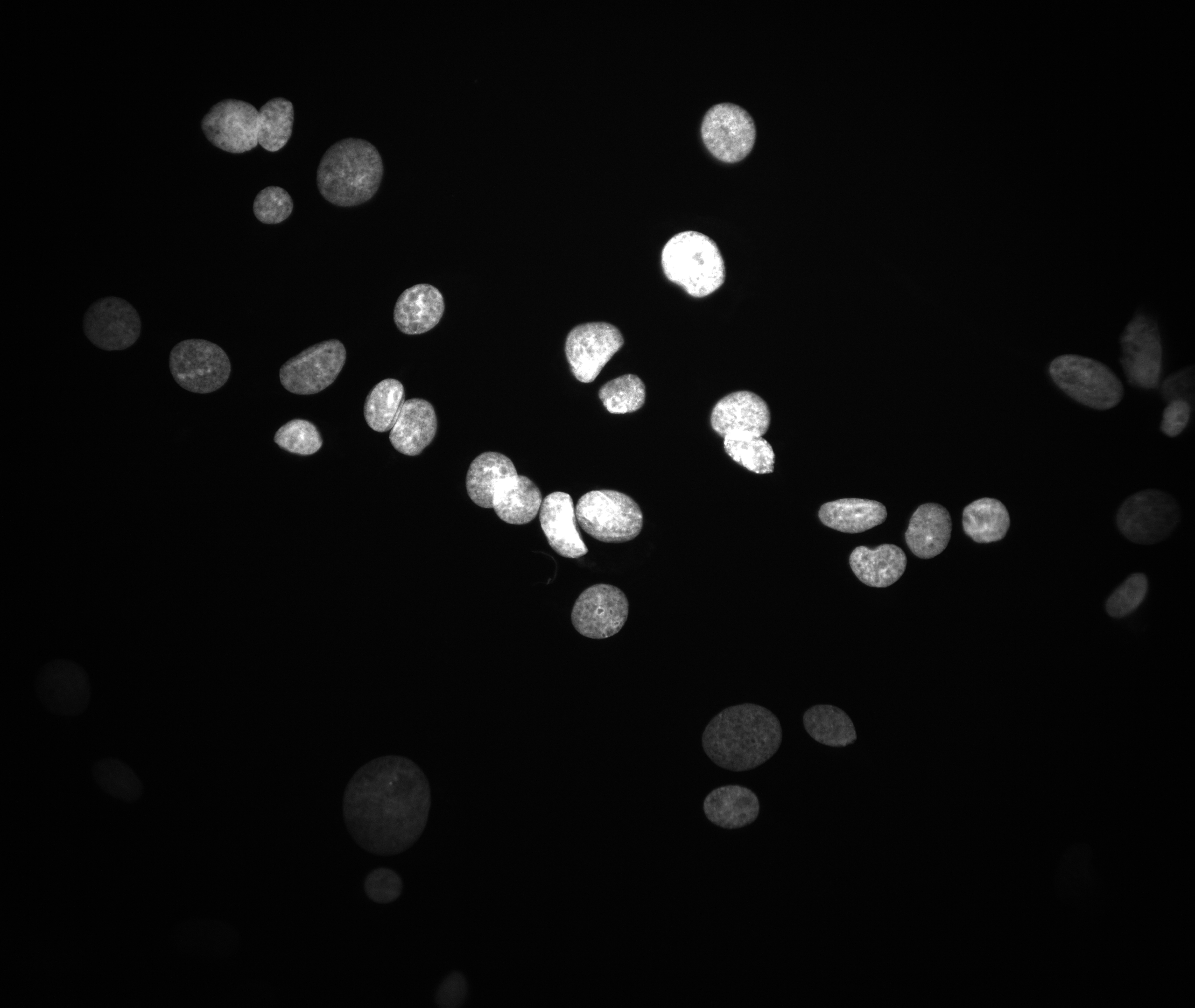

Supplement: Supplementary file 10 — Figure EV1 Source Data [file 44321_2025_347_MOESM10_ESM.zip › SD for Fig EV1/EV1A/EV1A (WT).tif]

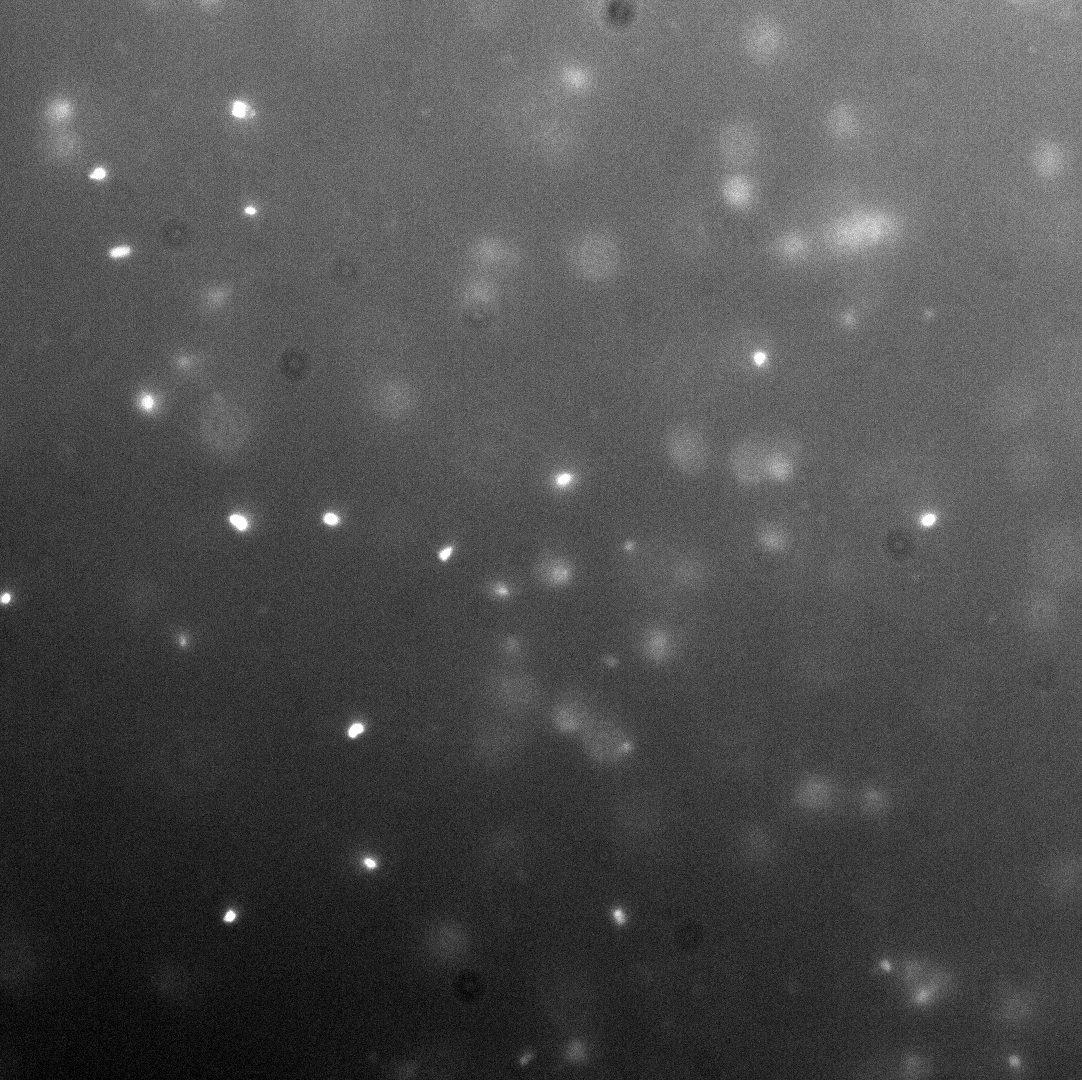

Supplement: Supplementary file 11 — Figure EV2 Source Data [file 44321_2025_347_MOESM11_ESM.zip › SD for Fig EV2/EV2C/control.tif]

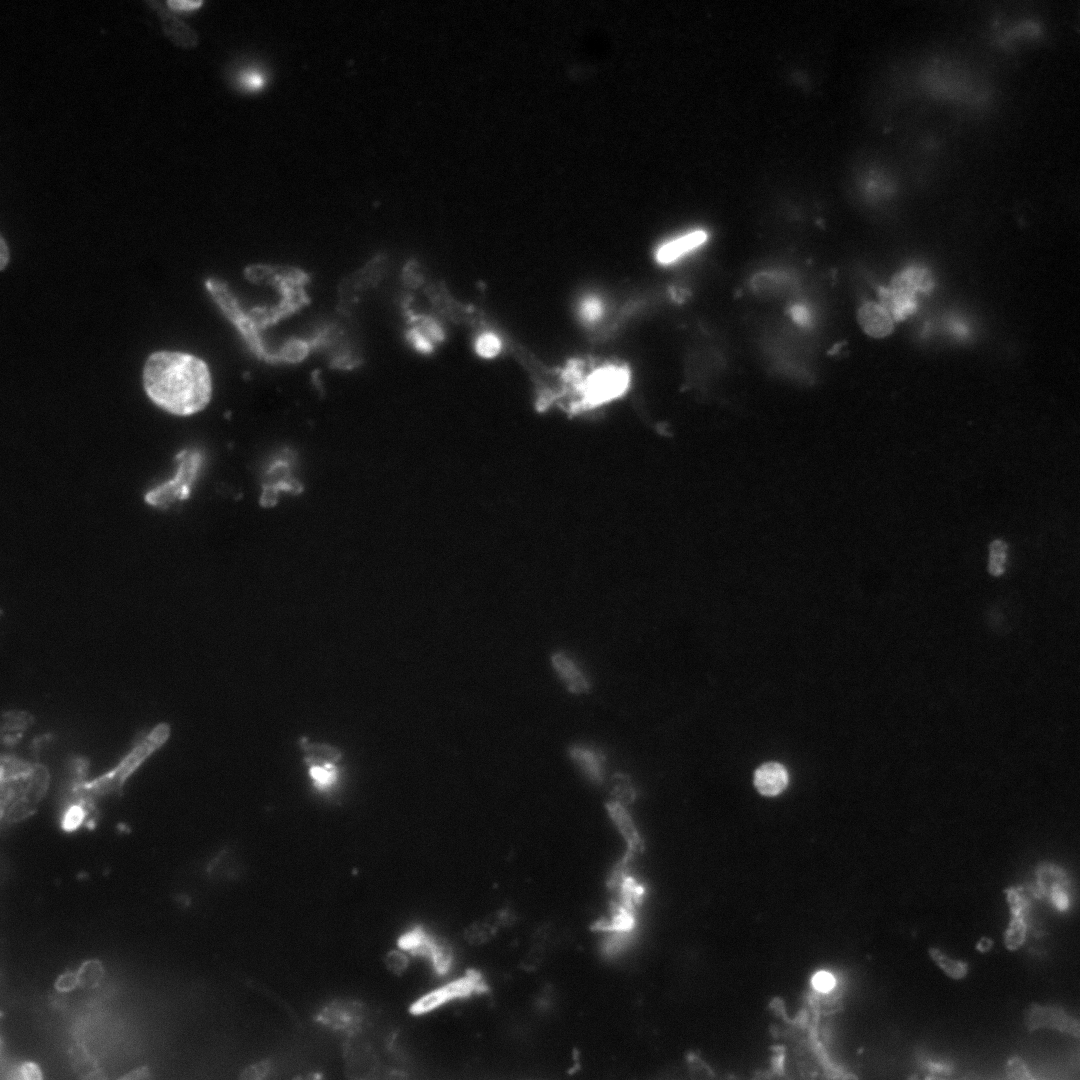

Supplement: Supplementary file 11 — Figure EV2 Source Data [file 44321_2025_347_MOESM11_ESM.zip › SD for Fig EV2/EV2C/Lysozyme (LYZ).tif]

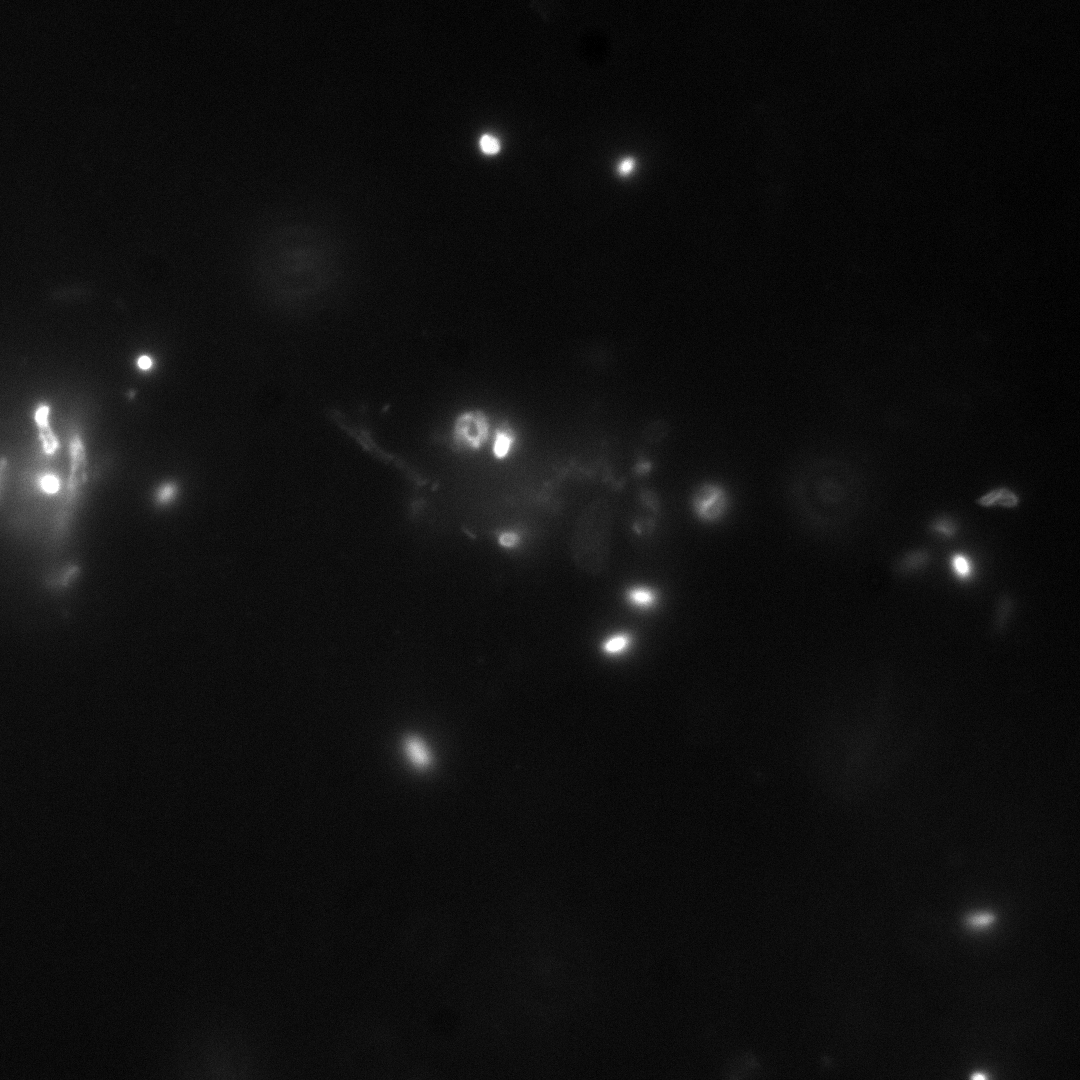

Supplement: Supplementary file 11 — Figure EV2 Source Data [file 44321_2025_347_MOESM11_ESM.zip › SD for Fig EV2/EV2C/Lysozyme:Lactoferrin (LYZ:LFN).tif]

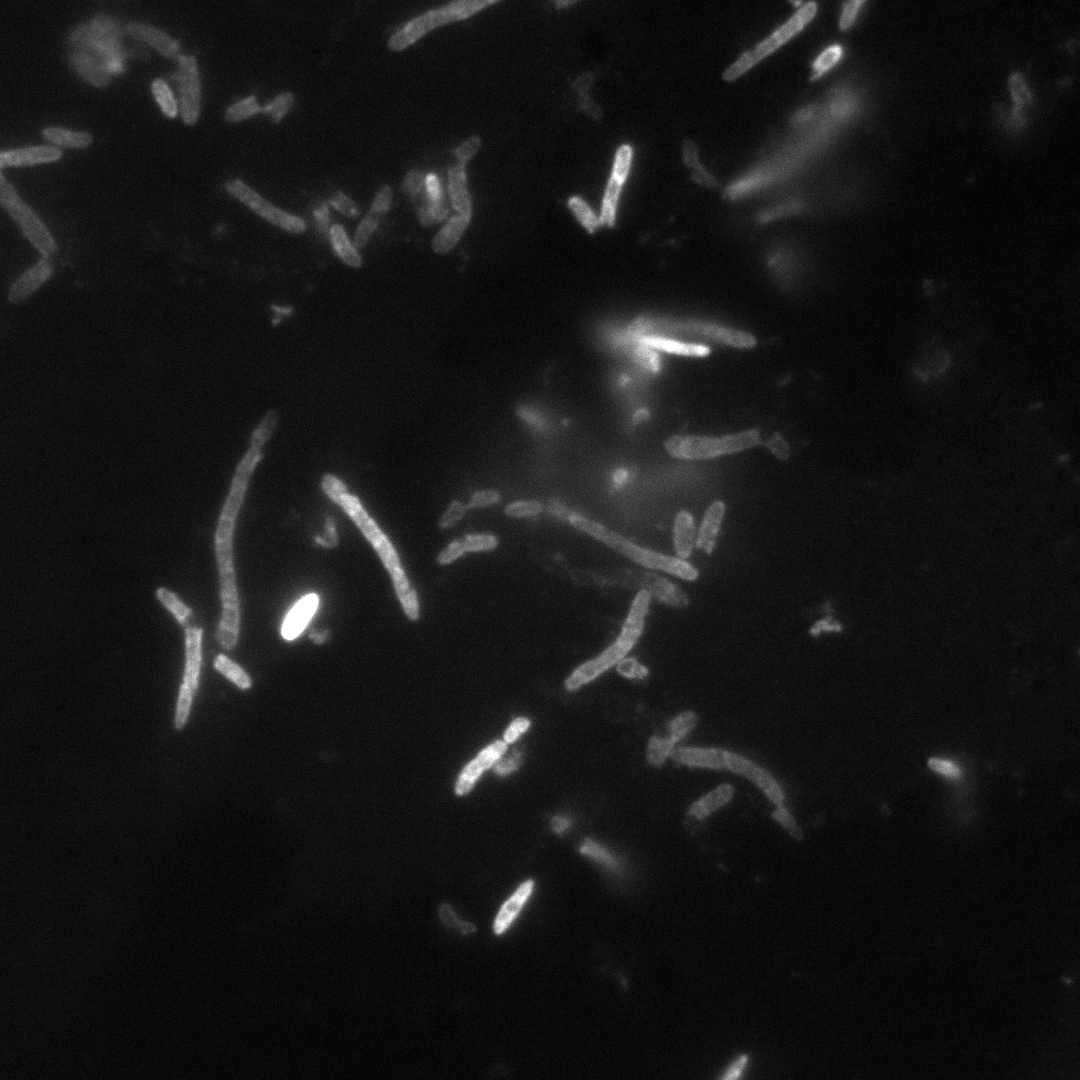

Supplement: Supplementary file 11 — Figure EV2 Source Data [file 44321_2025_347_MOESM11_ESM.zip › SD for Fig EV2/EV2C/Lactoferrin (LFN).tif]

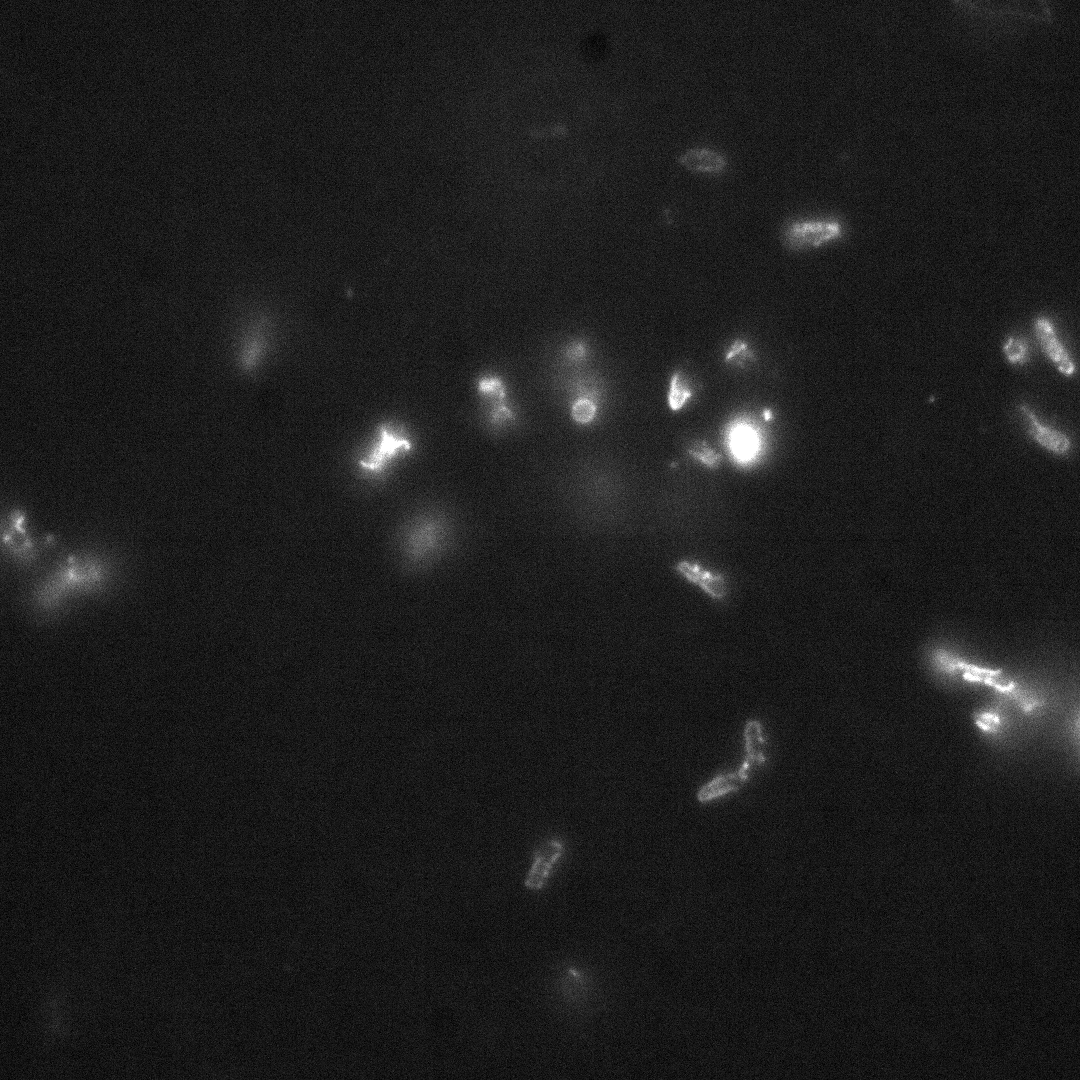

Supplement: Supplementary file 11 — Figure EV2 Source Data [file 44321_2025_347_MOESM11_ESM.zip › SD for Fig EV2/EV2C/Lysozyme:EDTA (LYZ:EDTA).tif]

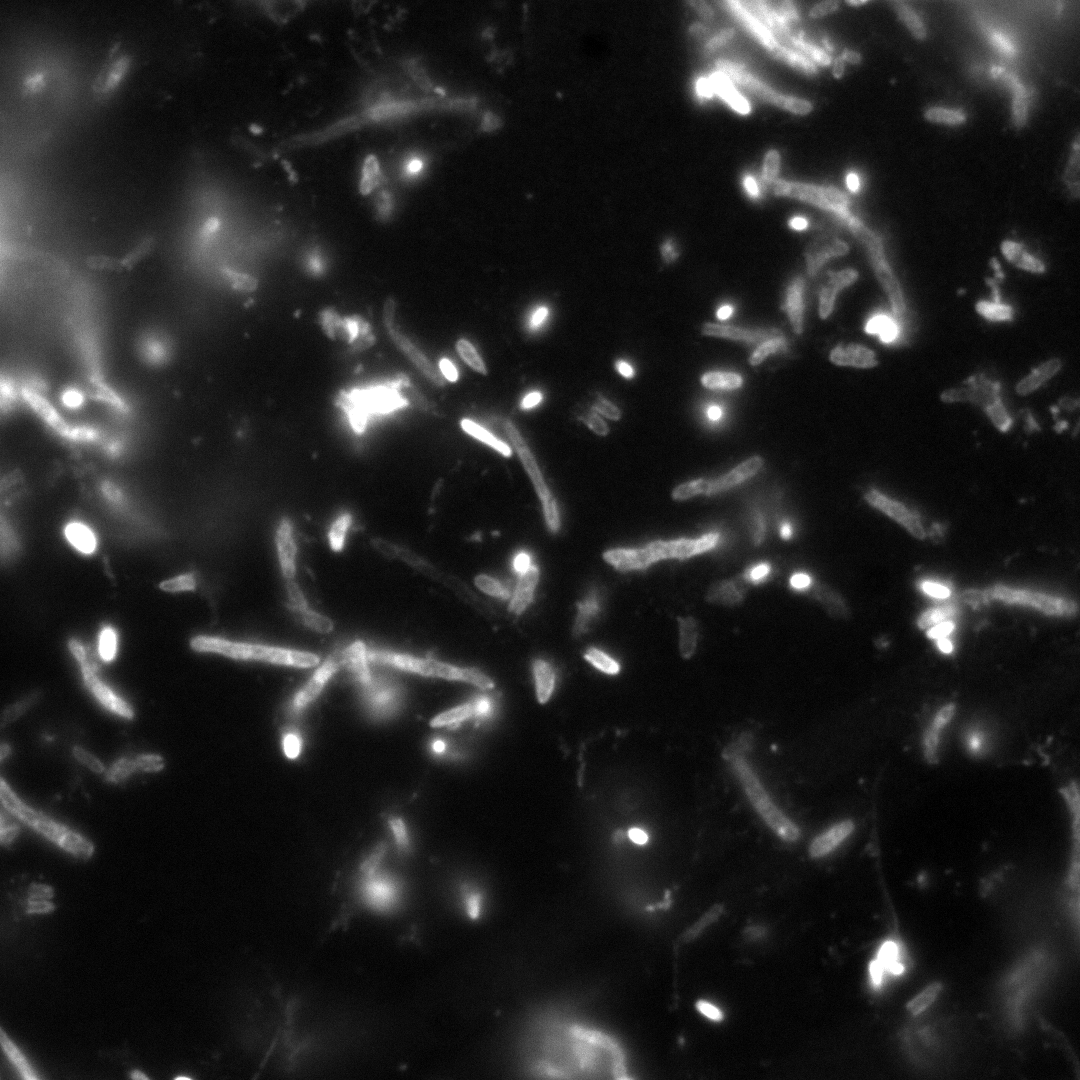

Supplement: Supplementary file 11 — Figure EV2 Source Data [file 44321_2025_347_MOESM11_ESM.zip › SD for Fig EV2/EV2C/Untreated.tif]

Fig EV4C

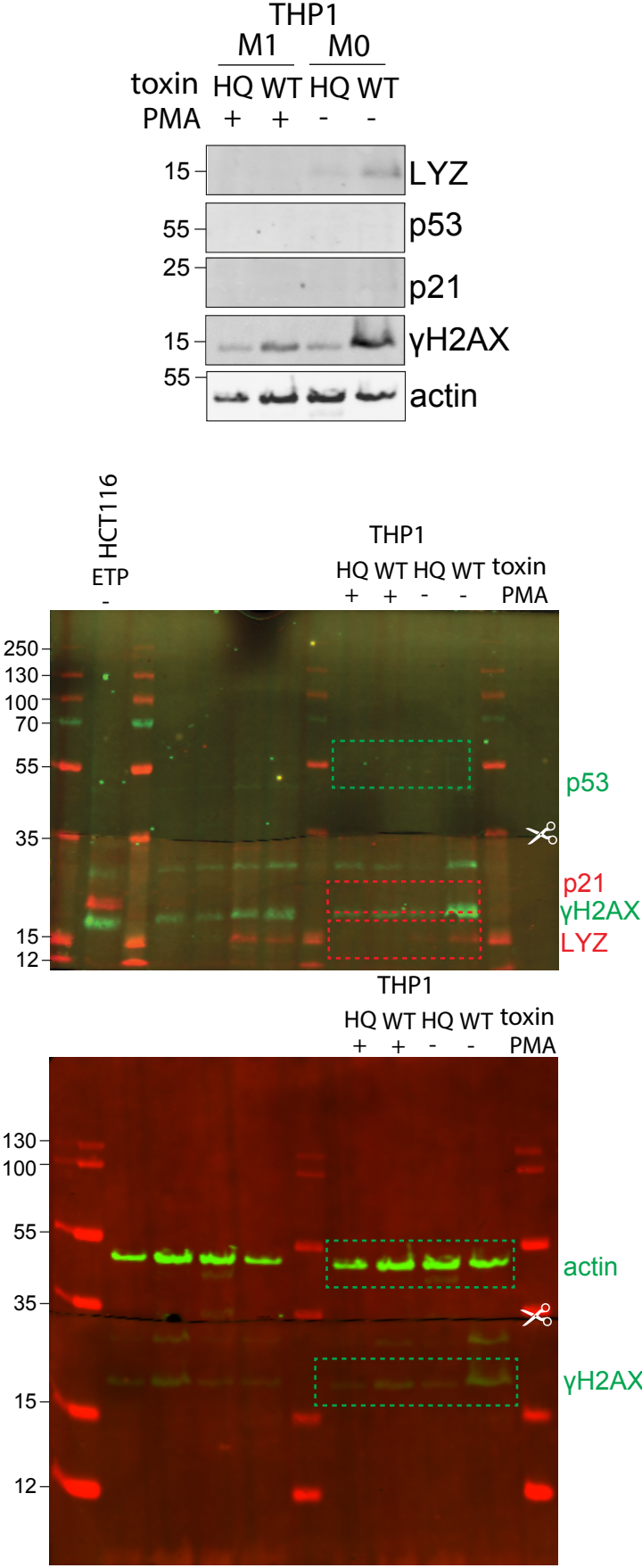

Supplement: Supplementary file 13 — Figure EV4 Source Data [file 44321_2025_347_MOESM13_ESM.zip › SD for Fig EV4/EV4C/EV4B_SD.pdf]

Fig EV4B

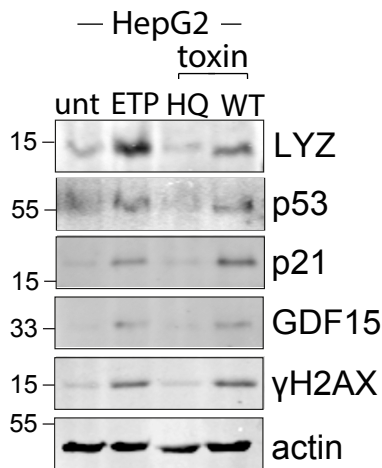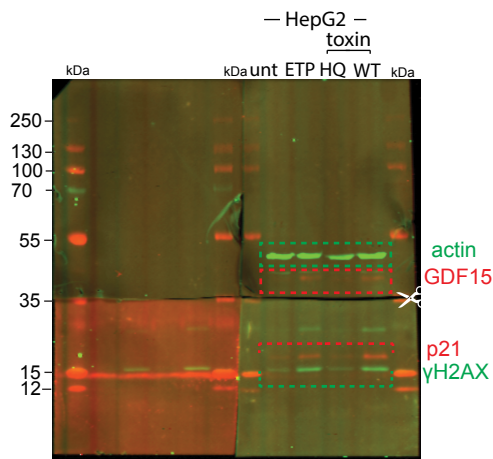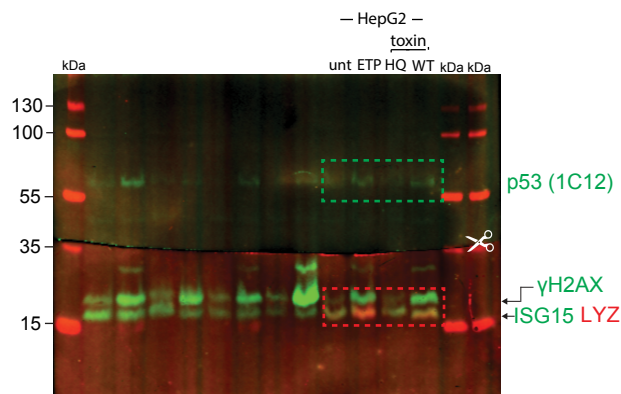

Supplement: Supplementary file 13 — Figure EV4 Source Data [file 44321_2025_347_MOESM13_ESM.zip › SD for Fig EV4/EV4B/EV4B_SD.pdf]
