# Supplementary material for: Typhoid toxin of Salmonella Typhi elicits host antimicrobial response during acute typhoid fever
Source: EMBO Mol Med. 2025 Dec 1;18(1):187–216. doi: 10.1038/s44321-025-00347-8 (PMC12808722; doi:10.1038/s44321-025-00347-8)

READ ME

Workflow for immunoblot figure assembly

The image below provides an example workflow for generating immunoblot figures.

1. Raw data was captured in LiCor Image Studio 6.1 software, which showed green and red channels.
2. The desired channel was selected on Image Studio (red selected in example below).
3. The channel was made greyscale (black on white) and exported as a TIFF file.
4. Bands of interest were cut using Adobe Photoshop and pasted into Adobe Illustrator for figure assembly.

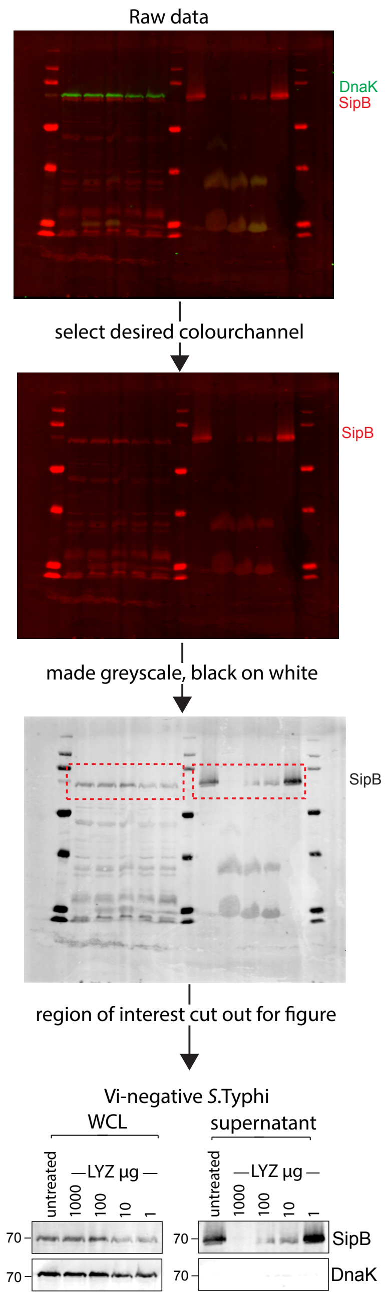

Supplement: Supplementary file 7 — Source data Fig. 4 [file 44321_2025_347_MOESM7_ESM.zip › SD for Fig 4/4E/READ ME.pdf]
