# Supplementary material for: Typhoid toxin of Salmonella Typhi elicits host antimicrobial response during acute typhoid fever
Source: EMBO Mol Med. 2025 Dec 1;18(1):187–216. doi: 10.1038/s44321-025-00347-8 (PMC12808722; doi:10.1038/s44321-025-00347-8)
Supplement: Supplementary file 12 — Figure EV3 Source Data [file 44321_2025_347_MOESM12_ESM.zip › SD for Fig EV3/EV3C/EV3C_SD.pdf]

Fig EV3C

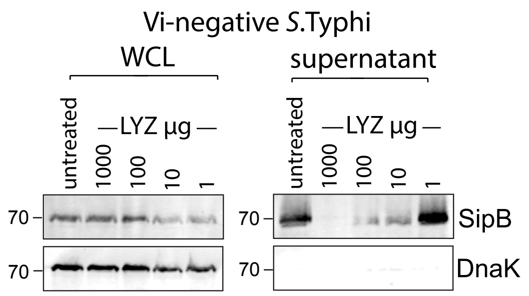

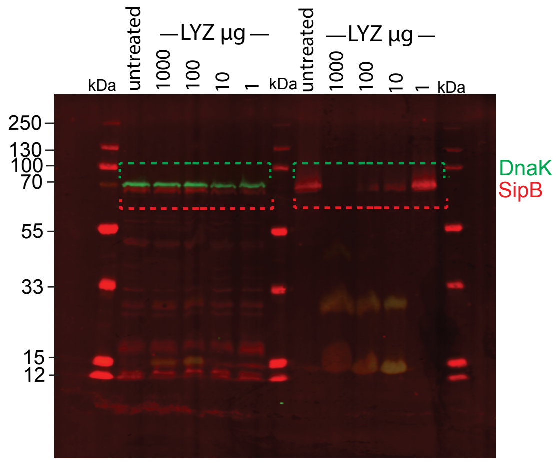

(i) Dashed boxes in green or red indicate bands excised for generating figure panels

(ii) Molecular weight markers are indicated in kDa

(iii) If appropriate, scissors indicate where immunoblots were cut to incubate with different antibodies
