# Supplementary material for: Typhoid toxin of Salmonella Typhi elicits host antimicrobial response during acute typhoid fever
Source: EMBO Mol Med. 2025 Dec 1;18(1):187–216. doi: 10.1038/s44321-025-00347-8 (PMC12808722; doi:10.1038/s44321-025-00347-8)
Supplement: Supplementary file 13 — Figure EV4 Source Data [file 44321_2025_347_MOESM13_ESM.zip › SD for Fig EV4/EV4A/EV4A.pdf]

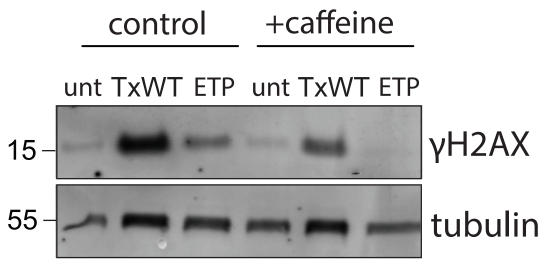

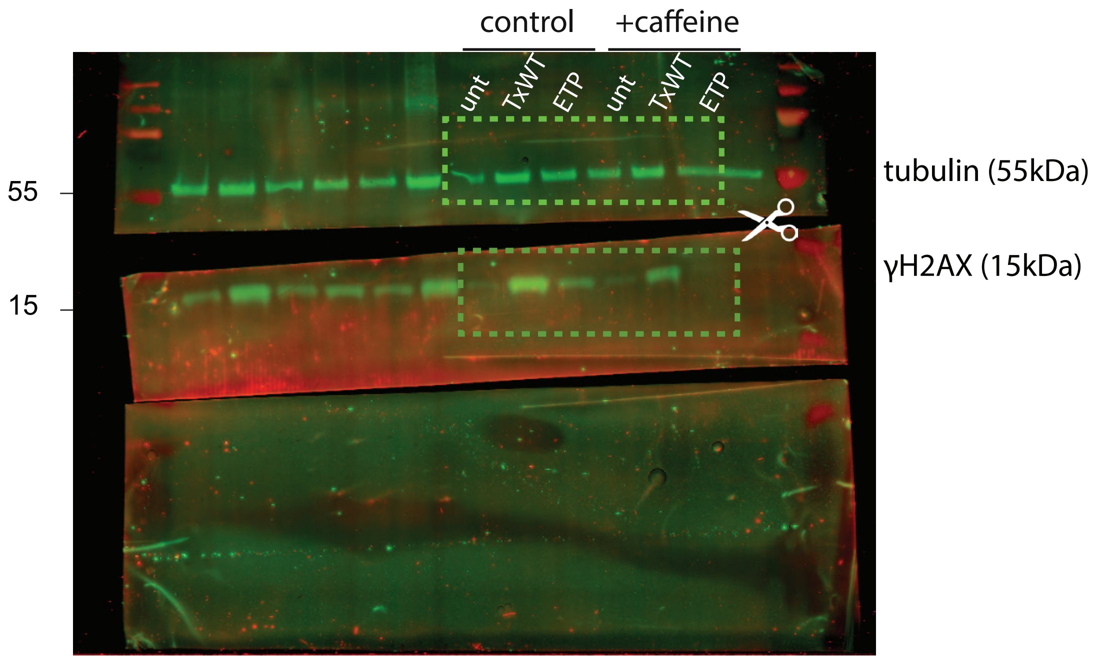

(i) Dashed boxes in green indicate bands excised for generating figure panels
(ii) For this blot, gels were cut prior to imaging
(iii) tubulin antibody (62204 - Invitrogen) ; γH2AX (05-636 - Millipore)
