# Supplementary material for: Typhoid toxin of Salmonella Typhi elicits host antimicrobial response during acute typhoid fever
Source: EMBO Mol Med. 2025 Dec 1;18(1):187–216. doi: 10.1038/s44321-025-00347-8 (PMC12808722; doi:10.1038/s44321-025-00347-8)
Supplement: Supplementary file 13 — Figure EV4 Source Data [file 44321_2025_347_MOESM13_ESM.zip › SD for Fig EV4/EV4A/READ ME.pdf]

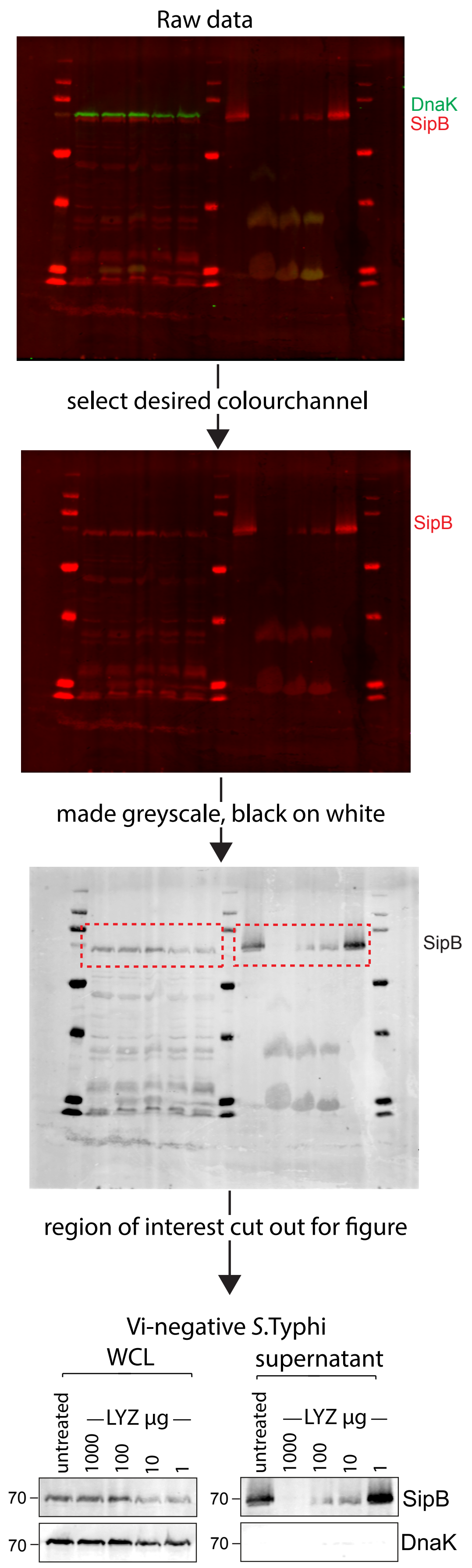
Raw data
DnaK
SipB
select desired colourchannel
SipB
made greyscale, black on white
SipB
region of interest cut out for figure
Vi-negative S.Typhi
WCL
supernatant
untreated
—LYZ µg—
1000
100
10
1
untreated
—LYZ µg—
1000
100
10
1
70
70
70
70
SipB
DnaK
