# Supplementary material for: Typhoid toxin of Salmonella Typhi elicits host antimicrobial response during acute typhoid fever
Source: EMBO Mol Med. 2025 Dec 1;18(1):187–216. doi: 10.1038/s44321-025-00347-8 (PMC12808722; doi:10.1038/s44321-025-00347-8)
Supplement: Supplementary file 14 — Expanded View Figures [file 44321_2025_347_MOESM14_ESM.pdf]

Expanded View Figures

Figure EV1. APOC3 expression in HepG2 liver cells treated with typhoid toxin.

(**A**) Fluorescence microscopy images of HepG2 intestinal cells, from three independent experiments, either untreated, treated with wild-type typhoid toxin (TxWT) or H160Q DNase-deficient toxin (TxHQ) for 2 h prior to imaging at 96 h of EdU (magenta) or APOC3 (yellow). DAPI-stained nuclear outlines shown. Scale bars: 50 µm. (**B**) Bar chart showing proportion of APOC3-positive cells ($n = 4$), or (**C**) EdU-positive HepG2 cells ($n = 3$), at 96 h. Circles indicate biological repeats. (**D**) Quantification of *Salmonella* CFUs following incubation with 50 mg/ml of purified APOC3 at 1 h, 2 h and 4 h ($n = 3$). Statistical significance: Welch's unpaired t-test for paired measures with unequal variances in (**B**, **C**), two-way ANOVA Sidak multiple comparisons (**D**) assessing 2 independent variables. Data are presented as mean ± SEM. Asterisks indicate significance: *$P < 0.05$, **$P < 0.01$, ***$P < 0.001$, ****$P < 0.0001$. No significance (ns). Exact *P* values in Appendix Table S1. Circles and n represent biological replicates. Experiments in EV2 linked to Fig. 2. Source data are available online for this figure.

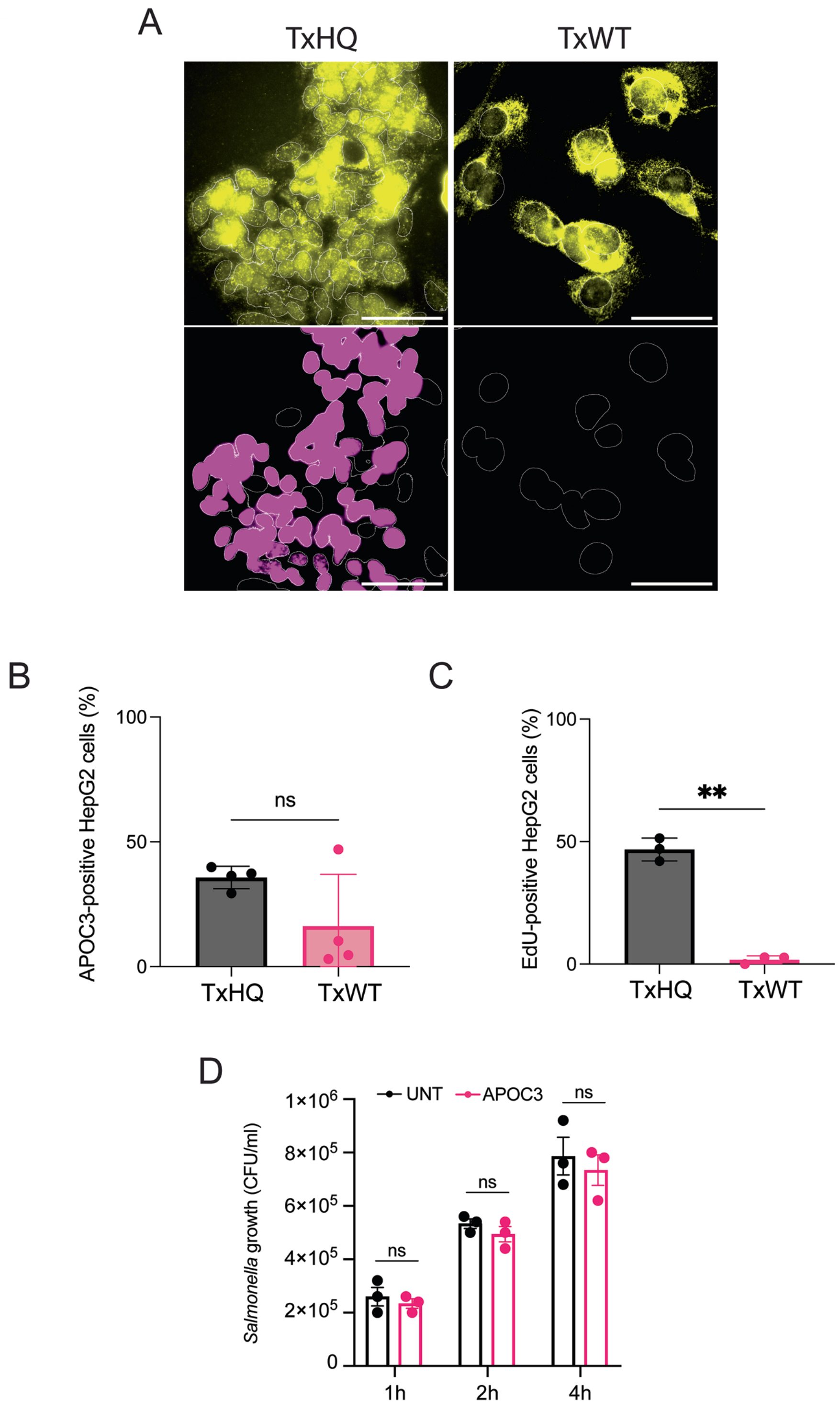
A
TxHQ
TxWT
B
APOC3-positive HepG2 cells (%)
100
50
0
ns
TxHQ
TxWT
C
EdU-positive HepG2 cells (%)
100
50
0
**
TxHQ
TxWT
D
Salmonella growth (CFU/ml)
UNT
APOC3
1×10^6
8×10^5
6×10^5
4×10^5
2×10^5
0
ns
1h
2h
4h

 © The Author(s)

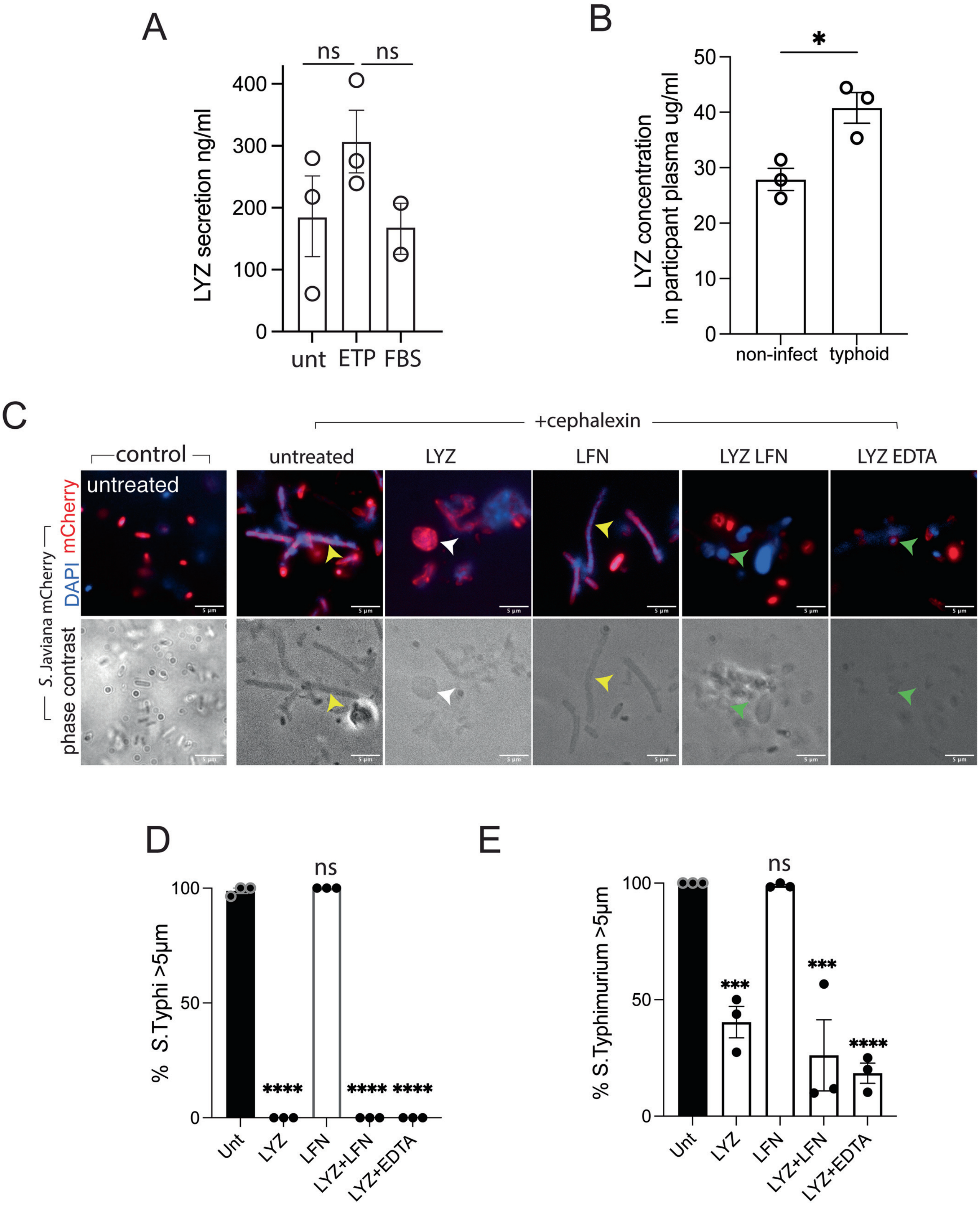
A
ns
ns
400
300
200
100
0
LYZ secretion ng/ml
unt
ETP
FBS
B
*
50
40
30
20
10
0
LYZ concentration in participant plasma ug/ml
non-infect
typhoid
C
+cephalexin
control
untreated
LYZ
LFN
LYZ LFN
LYZ EDTA
untreated
S.Javiana mCherry
DAPI mCherry
phase contrast
D
ns
100
50
0
% S.Typhi >5µm
****
**** ****
Unt
LYZ
LFN
LYZ+LFN
LYZ+EDTA
E
ns
100
50
0
% S.Typhimurium >5µm
***
***
****
Unt
LYZ
LFN
LYZ+LFN
LYZ+EDTA

Figure EV2. Spheroplast formation in response to LYZ.

(**A**) ELISA of LYZ using growth media from untreated or etoposide-treated (ETP) CACO2 cells at 96 h, or 10% FBS used to supplement growth media as control ($n = 3$). (**B**) ELISA of LYZ using plasma from human participants in TYGER study at baseline (non-infect) or at typhoid diagnosis following *S*. Typhi infection (typhoid). Circles represent participants and biological repeats ($n = 3$). (**C**) Fluorescence microscopy images of cephalexin-treated *S*. Javiana pFPV-mCherry treated with LYZ, LFN, LYZ-LFN or LYZ-EDTA, from three independent experiments, before imaging mCherry *Salmonella* and DAPI-staining by fluorescence microscopy (top panel) or phase contrast (bottom panel). Elongated bacteria >5 µm (yellow arrows), spheroplasts with loss of mCherry (green arrows), and spheroplasts with mCherry retention (white arrows). Untreated, LYZ, and LYZ/LFN images reused in Fig. 3G. Scale bars: 5 µm. Bar charts showing the proportion of long bacteria (>5 µm) following cephalexin treatment of (**D**) *S*. Typhi or (**E**) *S*. Typhimurium in the absence (unt) or presence of LYZ, LFN, LYZ and LFN, LYZ and EDTA ($n = 3$). Statistical significance: one-way ANOVA Tukey's multiple comparison (**A**) analysing all pairs of >3 groups: Welch's unpaired *t* test for paired measures with unequal variances in (**B**); one-way ANOVA with Brown–Forsythe (**D**, **E**) for unequal variances (>3 groups). Data are presented as mean ± SEM. Asterisks indicate significance: *$P < 0.05$, **$P < 0.01$, ***$P < 0.001$, ****$P < 0.0001$. No significance (ns). Exact *P* values in Appendix Table S1. Circles represent biological replicates. Experiments in EV2 linked to Fig. 3. Source data are available online for this figure

 © The Author(s)

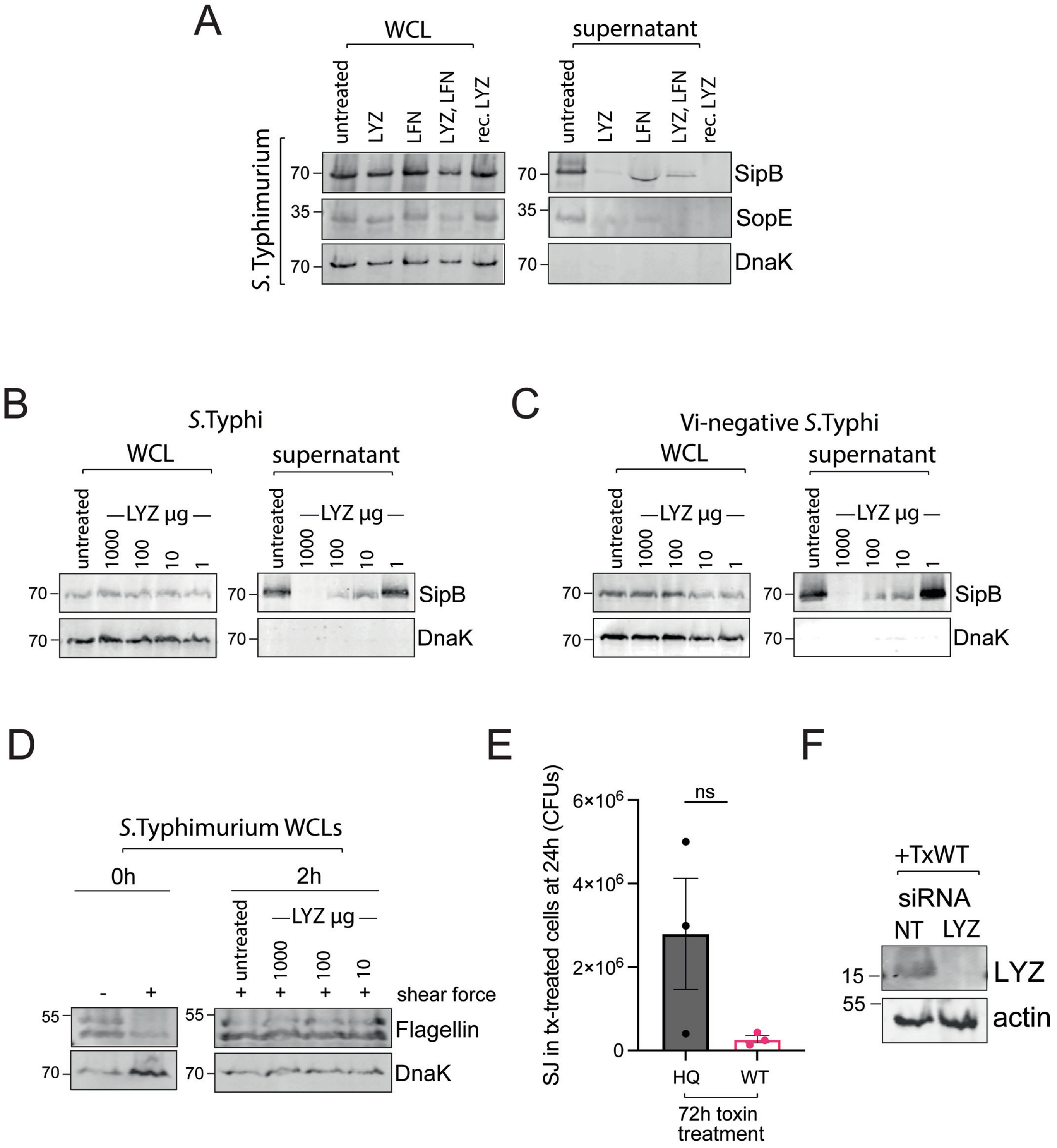
A
WCL
supernatant
untreated
LYZ
LFN
LYZ, LFN
rec. LYZ
S. Typhimurium
70
35
SipB
SopE
DnaK
B
S.Typhi
WCL
supernatant
untreated
LYZ µg
1000
100
10
1
SipB
DnaK
C
Vi-negative S.Typhi
WCL
supernatant
D
S.Typhimurium WCLs
0h
2h
shear force
55
Flagellin
DnaK
E
SJ in tx-treated cells at 24h (CFUs)
ns
6×10^6
4×10^6
2×10^6
0
HQ
WT
72h toxin treatment
F
+TxWT
siRNA
NT
LYZ
15
55
LYZ
actin

Figure EV3. Influence of LYZ on the T3SS of *Salmonella enterica.*

(**A**) Immunoblot of *S.* Typhimurium in LB broth either untreated, or cultured with 1 mg/ml endogenous LYZ, 100 µg/ml LFN, LYZ and LFN, or 1 mg/ml recombinant LYZ (rec. LYZ) for 2 h ($n = 2$). Whole cell lysates (WCLs) or supernatants immunoblotted with antibodies to virulence effectors SipB or SopE, or the intracellular loading control DnaK. MW in kDa, left. Whole cell lysates or supernatants from (**B**) *S.* Typhi BRD948 or (**C**) mutant Vi-deficient *S.* Typhi BRD948, cultured in LB only (untreated) or treated for 2 h with indicated concentrations of LYZ (n = 2). Antibodies to SipB or DnaK indicated. MW in kDa, left. (**D**) Export of *S.* Typhimurium FliC (Flagellin) to the outer membrane in the presence of indicated LYZ concentrations. *S.* Typhimurium were cultured to 0.5 OD_{600} in LB then either left untreated (−) or subjected to shear forces (+) to break flagella before incubating for 2 h with indicated concentrations of LYZ ($n = 2$). Whole cell lysates were immunoblotted with antibodies to flagellin or DnaK. MW in kDa, left. (**E**) *S.* Javiana (SJ) CFUs calculated on LB agar plates at 24 h post-infection from HCT116 cells already treated for 72 h with TxWT or TxHQ, ($n = 3$). (**F**) Immunoblot showing LYZ knockdown. HCT116 cells were transfected with non-targeting (NT) or LYZ siRNA for 48 h prior to treatment with TxWT and immunoblotting after 48 h (96 h total) ($n = 2$). Whole cell lysates immunoblotted with LYZ or actin antibodies. MW in kDa, left. Statistical significance: Welch's unpaired *t* test for paired measures with unequal variances in (**E**). Data are presented as mean ± SEM. Asterisks indicate significance: *$P < 0.05$, **$P < 0.01$, ***$P < 0.001$, ****$P < 0.0001$. No significance (ns). Exact *P* values in Appendix Table S1. Circles and n represent biological replicates. Experiments in EV2 linked to Fig. 4. Source data are available online for this figure

 © The Author(s)

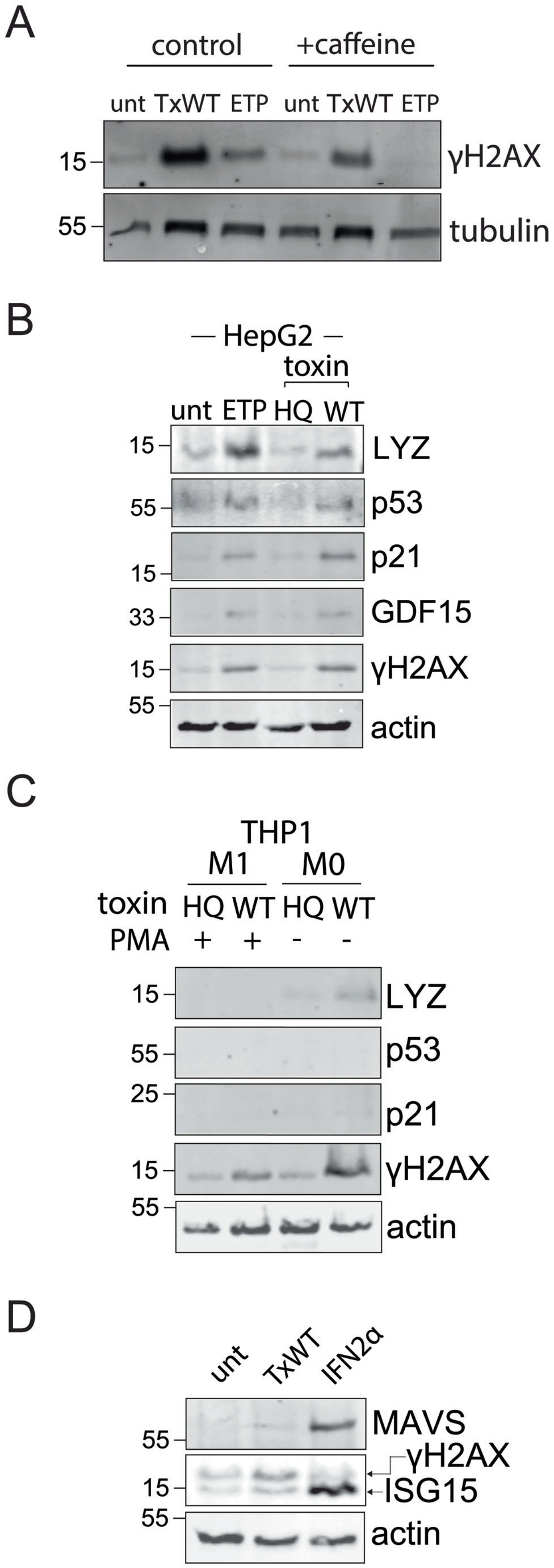
A
control
+caffeine
unt TxWT ETP unt TxWT ETP
15
γH2AX
55
tubulin
B
HepG2
toxin
unt ETP HQ WT
15
LYZ
55
p53
p21
15
33
GDF15
15
γH2AX
55
actin
C
THP1
M1
M0
toxin HQ WT HQ WT
PMA + + - -
15
LYZ
55
p53
25
p21
15
γH2AX
55
actin
D
unt
TxWT
IFN2α
MAVS
55
γH2AX
15
ISG15
55
actin

Figure EV4. LYZ expression in response to DDRs.

(**A**) Immunoblot showing the effect of caffeine on DDRs induced by TxWT or ETP. CACO2 cells were treated for 2 h with TxWT or ETP before addition of 10 mM caffeine for 48 h ($n = 2$). Whole cell lysates immunoblotted with antibodies to γH2AX and tubulin. MW in kDa, left. (**B**) LYZ expression and p53 responses in HepG2 liver epithelial cells at 72 h following no treatment (unt), or treatment with etoposide, TxHQ or TxWT ($n = 2$). Immunoblots performed with indicated antibodies. MW in kDa, left. (**C**) The same experiment as (**B**) performed with TxHQ or TxWT in THP1 macrophages differentiated to non replicate with addition of PMA (+), or in the precursor replicating THP1 monocyte form ($n = 2$). (**D**) Immunoblotting of interferon-stimulated genes MAVS and ISG15 in HCT116 cells at 72 h when untreated (unt), treated with TxWT or IFN2α as control ($n = 2$). Antibodies indicated, right. MW in kDa, left. Experiments in EV4 linked to Figs. 5 and 6. Source data are available online for this figure

 © The Author(s)
